# Induction of mitochondria mediated apoptosis in human ovarian cancer cells by folic acid coated tin oxide nanoparticles

**DOI:** 10.1371/journal.pone.0258115

**Published:** 2021-10-01

**Authors:** Demiana H. Hanna, Gamal R. Saad

**Affiliations:** Faculty of Science, Department of Chemistry, Cairo University, Giza, Egypt; VIT University, INDIA

## Abstract

**Purpose:**

This study aims to prepare folic acid coated tin oxide nanoparticles (FA-SnO_2_ NPs) for specifically targeting human ovarian cancer cells with minimum side effects against normal cells.

**Methods:**

The prepared FA-SnO_2_ NPs were characterized by FT-IR, UV-vis spectroscopy, XRD, SEM and TEM. The inhibition effects of FA-SnO_2_ NPs against SKOV3 cancer cell were tested by MTT and LDH assay. Apoptosis induction in FA-SnO_2_ NPs treated SKOV3 cells were investigated using Annexin V/PI, AO/EB and Comet assays and the possible mechanisms of the cytotoxic action were studied by Flow cytometry, qRT-PCR, Immunohistochemistry, and Western blotting analyses. The effects of FA-SnO_2_ NPs on reactive oxygen species generation in SKOV3 cells were also examined. Additionally, the safety of utilization FA-SnO_2_ NPs were studied in vivo using Wister rats.

**Results:**

The obtained FA-SnO_2_ NPs displayed amorphous spherical morphology with an average diameter of 157 nm and a zeta potential value of -24 mV. Comparing to uncoated SnO_2_ NPs, FA-SnO_2_ NPs had a superior inhibition effect towards SKOV3 cell growth that was suggested to be mediated through higher reactive oxygen species generation. It was showed that FA-SnO_2_ NPs increased significantly the % of apoptotic cells in the sub- G1 and G2/M phases with a higher intensity comet nucleus in SKOV3 treated cells. Furthermore, FA-SnO_2_ NPs was significantly increased the expression levels of P53, Bax, and cleaved Caspase-3 and accompanied with a significant decrease of Bcl-2 in the treated SKOV3 cells.

**Conclusion:**

Overall, the results suggested that an increase in cellular FA-SnO_2_ NPs internalization resulted in a significant induced cytotoxicity in SKOV3 cancer cells in dose-dependent mode through ROS-mediated cell apoptosis that may have occurred through mitochondrial pathway. Additionally, the results confirmed the safety of utilization FA-SnO_2_ NPs against living systems. So, FA-SnO_2_ NPs with a specific targeting moiety may be a promising therapeutic candidate for human ovarian cancer.

## 1. Introduction

Ovarian carcinoma is classified as the seventh most common cause of cancer mortality in women worldwide and the principal cause of death from gynecologic malignancies **[1]**. Currently, the primary therapy of ovarian cancer is based on a combination of optimum surgical therapy with platinum-based chemotherapy **[2]**. Despite the advances afforded to minimize the toxic and harmful effects toward health tissues of traditional chemotherapy, there is a need to develop an effective and safer treatment for cancer diseases.

Recently, the application of nanotechnology in medicine have attracted much interest as a potential alternative therapy in oncology field for decreased side effects of drug chemotherapy **[3,4].** Some previous studies demonstrated that, nanoparticles (NPs) have different physical and chemical properties, availability, and biocompatibility, resulting in enhanced selectivity against different cancer cells with a fewer side effects against healthy tissues **[5–11].** However, the potential applications of these NPs for therapeutic purposes in different cancer types can be highly effective through targeting different receptors of cancer surfaces.

It is known that NPs are commonly internalized inside the cells through receptor- mediated endocytosis pathway via coating of NPs surfaces with specific targeting ligands or through phagocytosis process **[12]**. NPs are tiny materials having size ranges from 1 to 100 nm and may be functionalized with organic molecules. Thus, modification of NPs surfaces with specific targeting ligands resulted in highly accumulated tagged NPs inside the receptor containing cells with higher efficacy.

Among these, folic acid (FA) has a natural affinity towards membrane- folate receptors (FRs), which is poorly expressed in normal cells and over expressed in different cancer cells, especially ovarian cancer cells. In addition, FRs mediate folate uptake from outside of the cell to the intracellular compartment through endocytosis. Besides, FA has particular advantages, as it processes high stability, relatively of low cost, tissue permeability and poor immunogenic **[13,14]**. Consequently, several studies have found that the circulating FA coated NPs were highly capable of inducing apoptosis in different cancer cells than uncoated NPs **[15–19]**.

Tin oxide nanoparticles (SnO_2_ NPs), are considered as one of the most significant nanoparticles, which have strong chemical and physical interactions, biocompatibility, small functional temperature, and thermal stability up to 500˚C **[20].** Thus, SnO_2_ NPs have used in different application, such as solar cells, catalysts, gas sensors, lithium-ion batteries, and others **[21].** Recently, SnO_2_ NPs have more extensive use in biomedicine field, where some studies have shown that SnO_2_ NPs have antioxidant and antibacterial activities **[22]**. Moreover, recent studies have revealed the oxidative stress mediated cytotoxicity of SnO_2_ NPs in different human cancer cell lines such as oral cancer cells **[23]**, colorectal and lung cancer cells **[24]**, and breast cancer **[25]**. However, an earlier report has mentioned the potent toxic effects in hepatocellular carcinoma after exposure to SnO_2_ NPs without referring to their toxicity mechanisms **[26]**.

Oxidative stress is considered to be one of the most toxicity mechanisms of nanoparticles that stimulate series of cellular events resulting in DNA damage and apoptosis **[27]**. Nanoparticles can cause apoptosis in different cancer cells through activating apoptotic signaling through extrinsic pathway (death receptors), and or intrinsic pathway (mitochondria) **[28]**. The intrinsic pathway is stimulated through the proteins of Bcl2 family, which involves anti-apoptotic such as Bcl-2 and Bcl-XL, and pro-apoptotic proteins as Bax and Bak **[29]**. DNA damage or cellular stress can stimulate activation of tumor suppressor protein (p53), resulting in induction of the intrinsic apoptosis pathway through stimulation loss of mitochondrial integrity and activation of proapoptotic proteins as well as inactivating the antiapoptotic proteins **[30]**. Successively, the mitochondrial dysfunction resulting in activation of caspases that can cleaves the key death substrates in the cell to produce many of the cellular and biochemical events of apoptosis **[30]**. In addition, the activated p53 protein can also mediated apoptosis through arresting the cells at G1 **[31]**, and or G2/M phases **[32].**

Since the potential toxicity of SnO_2_ NPs against human ovarian cancer has not been studied yet, our current study was designed for assessment of the in vitro toxicity of SnO_2_ NPs and FA-SnO_2_ NPs against a human ovarian cancer cell line (SKOV3), along with exploring their toxicological mechanisms of apoptosis induction in SKOV3 cells. Therefore, the present study aims to prepare SnO_2_ NPs, by a simple sol-gel method, and SnO_2_ NPs coated with FA, where FA acts as a specific targeting ligand for SKOV3 cancer cell. The prepared nanoparticles were characterized by different analysis methods (FT-IR, UV-vis spectroscopy, XRD, SEM and TEM) and then evaluated their potential cytotoxic effects on proliferation, morphological changes, oxidative stress, and apoptosis induction in SKOV3 cells via various assays (MTT Annexin V/PI, AO/EB and Comet assays). Moreover, their cellular and molecular mechanisms of apoptosis induction were identified via different techniques (cell cycle arrest, qRT-PCR, western blotting analysis and immunohistochemistry), along with in vivo examination for the toxicity of FA- SnO_2_ NPs against healthy tissues in adult male Wistar rats.

## 2. Materials and methods

### 2.1 Chemicals and reagents

SnCl_4_.5H_2_O, CH_3_OH and folic acid were obtained from Sigma Aldrich, Germany, and used as received. All other used chemicals and reagents were of chemical grade and commercially accessible.

### 2.2 Preparation of SnO_2_ NPs and FA-SnO_2_ NPs

Firstly, SnO_2_ NPs were prepared using sol gel method as described before, **[22,33]**. Briefly, 3.5 g of SnCl_4_.5H_2_O was dissolved in 100 ml of CH_3_OH. Then under vigorous stirring, an aqueous ammonia solution (4.0 ml) was added drop-wisely to SnCl_4_.5H_2_O solution. After that, the resulting gel was filtered, washed with methanol and dried at 80°C in an oven for about 5 h. Finally, the resulting powder was calcined at 400°C for 2 h in an oven and the resulted colored powder tin oxide nanoparticles (SnO_2_ NPs) were collected. Secondly, post-synthesis step for the preparation of FA-SnO_2_ NPs was followed as described before **[15]**. About 100 mg of the prepared SnO_2_ NPs were dispersed in distilled water (25.0 ml) and sonicated for 10 min (T25 IKA digital Ultra-Turrax Disperser, Germany). Folic acid (FA, 10 mg) was dissolved in sodium hydrogen carbonate solution (5.0 ml, 0.1M) with adjustment pH to 5.5 at room temperature. Then, 5.0 ml of the prepared FA solution was added gradually to 25 ml of the suspension SnO_2_ NPs. The reaction was slightly shaken at room temperature for 24h. Finally, the obtained product (mixture) was dialyzed against distilled water for 48h using dialysis membrane (3500 MW, Spectra/Por, USA) for removing the residue of unreacted FA and the obtained FA coated tin oxide nanoparticles (FA-SnO_2_ NPs) were collected after 48h of freeze-drying using (Edwards Modulyo Freeze Dryer, England).

### 2.3 Physicochemical characterization

The ultraviolet-visible (UV-vis) spectrum for the FA, SnO_2_ NPs, and FA-SnO_2_ NPs was recorded in the absorption ranges from 200–700 nm using Shimadzu, UV- 3101PC UV-vis-NIR spectrophotometer.

The Fourier transform infrared (FT-IR) spectra of the FA, SnO_2_ NPs, and FA-SnO_2_ NPs were taken on FT-IR spectrophotometer (Varian, 640-IR, USA). The obtained spectra were recorded in the wave number region of 4000–500 cm^-1^ at a resolution of 4 cm^-1^ with a KBr pellet.

Powder X-ray Diffraction (PANalytical, X’Pert Pro, Netherlands) was employed to identify the crystalline structure of SnO_2_ NPs and FA-SnO_2_ NPs.

The mean particle size and zeta potential measurements of SnO_2_ NPs, and FA-SnO_2_ NPs were recorded via a particle size molecular measurement apparatus (Malvern Instruments Ltd., Nano- Sight NS500, Malvern, UK).

The morphology and particle size of SnO_2_ NPs, and FA-SnO_2_ NPs were detected using Scanning Electron Microscope (SEM, JEOL JSM-67001, Japan) and Transmission Electron Microscopy (TEM, JEOL JEM-2100, Japan).

### 2.4 Cell culture

The human ovarian cancer cell line (SKOV3 cells) was purchased from the American Type Culture Collection (ATCC). These cells were grown in Minimum Essential Medium Eagle (MEM), appended with 1.0% mixture of two antibiotics, (streptomycin and penicillin) and fetal bovine serum (10%), and kept at 37°C in an incubator using a 5% CO2 atmosphere. Using trypsin–EDTA, cells were trypsinized for collecting of the monolayer cells and then sub-cultured in culture flasks for maintenance and using as required in the following experiments.

### 2.5 Cytotoxicity assay using MTT method

The cytotoxic effect of SnO_2_ NPs and FA-SnO_2_ NPs against SKOV3 cells was assessed using MTT (3-(4,5-dimethyl-2-thiazolyl)-2,5-diphenyl-2H-tetrazolium bromide) assay as described previously **[15,34]**. This assay is a colorimetric assay that can detect the percentages of metabolically active cells. Briefly, 1×10^4^ of SKOV3 cells were seeded in each 96-well plate in the growth media (MEM) for overnight incubation at temperature 37°C. Then the cells were treated in triplicates with different concentrations (12.5, 25, 50, 100 μg/ml) for each of the two tested samples (SnO_2_ NPs and FA-SnO_2_ NPs) for 24 h incubation temperature 37°C. Furthermore, we examined the cell morphology of control and treated cells with an inverted microscope (Leica, DMI3000B) for checking any physical marks of toxicity such as rounding, partial or complete damage of the monolayer, cell granulation, and shrinkage. After that, the treated cells and untreated cells (control), were washed and treated with 20 μl of MTT solution (5 mg/ml in PBS) for 4h at 37°C for allowing metabolism of MTT (MTT will enter the mitochondria of the viable cells where it is reduced by succinate dehydrogenase (mitochondrial enzyme) into an insoluble dark purple formazan product, which is directly proportional to the number of viable cells. Furthermore, 200 μl of dimethyl sulfoxide (DMSO) was added to the cells with shaking (150 rpm for 5 minutes), for extraction of the formazan crystal (MTT metabolic product). Finally, the absorbance was measured at 570 nm using a microplate reader instrument (Bio-Rad, USA). The effect of the concentration of tested materials on the proliferation of human SKOV3 cells was expressed as the % cell viability and cytotoxicity %, using the following formulae **[35]**:

Cellviability%=[(Ae‐Ab)/(Ac‐Ab)]x100 (1)

Cytotoxicity%=100‐Viability% (2)

where Ae is the optical density of tested samples, Ac is the optical density of control, and Ab is the optical density of blank. All experiments of each tested sample were performed in triplicateso.

The concentration of required SnO_2_ NPs and FA-SnO_2_ NPs for a 50% inhibition of viability (IC50) was determined for incubation time, 24 h.

An IC_50_ which has a value less than 20 μg/ml of the tested samples was stated as an active sample against cancer cells, according to the standard National Cancer Institute (NCI) principles **[36]**.

### 2.6 Lactate dehydrogenase leakage test (LDH)

The cytotoxic effect of SnO_2_ NPs and FA-SnO_2_ NPs against SKOV3 cells was further estimated by a standard lactate dehydrogenase (LDH) release assay. Since LDH enzyme will outflow into the tested culture medium after cell death. The activities of LDH enzyme in the treated SKOV3 cells which were earlier incubated with IC_50_ values of SnO_2_ NPs and FA-SnO_2_ NPs for 24 h (evaluated from the MTT assay) and untreated cells (control) were measured spectrophotometrically at 340 nm in the culture medium and the cellular lysates, as earlier described **[37]**. Briefly, SKOV3 ovarian cancer cells were collected, located in 96-well plates at a density of 2 x 10^4^ cells per well, and incubated overnight before treatment with the tested samples (SnO_2_ NPs and FA-SnO_2_ NPs). After that, we removed 40 μl of the supernatant and placed it in a fresh well 96-well plate for measuring the released amount of LDH. Besides, the original plates were refilled by Triton X100 (40 μl) for assessing the concentration of total LDH. After that, we added pyruvic acid (100 μl) into each of the tested plates which having the supernatant only. Then, 100 μl of reduced β-NADH in potassium phosphate buffer pH 7.5, was distributed into the wells of plates. The loss of NADH to NAD+ depended on changing pyruvate into lactate which was detected via decreasing the measured absorbance at 340 nm using an ELISA microplate reader (Bio-Rad, Model 550, USA). This technique was repeated but with adding the total cell lysate (40 μl) of the original plates for assessment of the total LDH concentrations. The percentage of LDH leakage into the two tested culture medium was calculated using Eq 3 as follow:

LDHactivity(%)=activityofthesupernatant/totalactivityx100 (3)

where total LDH activity = LDH activity of cell lysate + LDH activity of the supernatant

### 2.7 Detection of apoptosis induction

#### 2.7.1 Quantification of apoptosis extent using Annexin V/PI assay

Annexin V/PI assay can distinguish apoptotic and necrotic cells using both Annexin V-fluorescein isothiocyanate (FITC) and propidium iodide (PI) stains, established on translocation of phosphatidylserine (PS) from the inner plasma membrane into outer cell surface in apoptotic cells where it can particularly bind with a fluorescent Annexin V -FITC, also PI can distinguish permeabilized plasma membrane of necrotic cells. Thus the percentages of apoptosis/necrosis cells in untreated SKOV3 cells and the treated SKOV3 ovarian cancer cells that were previously treated with an IC_50_ concentration of SnO_2_ NPs and FA-SnO_2_ NPs for 24 h, was assessed by an Annexin V-FITC detection kit I (BD Biosciences) as previously mentioned **[38,39].** Briefly, SKOV3 cells were harvested and placed in a culture plate (6-well) such that, each well has 1 x10^6^ cells per well and allowed for incubation overnight for cell adhesion and proliferation. After that, the SKOV3 cells were treated with SnO_2_ NPs and FA-SnO_2_ NPs for 24 h. Furthermore, the treated and control cells were trypsinized, centrifuged, and washed two times using cold PBS. Then, the washed cells were added to the Binding Buffer of Annexin V (1X), and then 100 μl of this cell solution was transferred into a culture tube (5 μl) to allow the incubation with Annexin V-FITC (5 μl) and propidium iodide (PI, 5 μl) at room temperature for 15 min in the dark. finally, the stained cells were diluted with the binding buffer (1X, 400 μl) and were investigated for the existence of apoptotic cells (early and late) and also necrotic cells using a flow cytometer (BD Biosciences, BD FACSCalibur™). The results are displayed as the rate of apoptosis which equals the percentages of early plus the percentages of late apoptotic cells.

#### 2.7.2 Morphological investigation using fluorescence microscopy

Induction of Apoptosis in SKOV3 cells as a result of treatment with SnO_2_ NPs and FA—SnO_2_ NPs was studied using acridine orange/ethidium bromide (AO/EB) stain assay. The AO/EB stain is considered to be a viability stain that detects apoptotic cell percentages. The intact membranes of apoptotic cells (normal and early), will allow penetration of AO into it and binds with their DNA content, so it gives green fluorescence, while the damaged membranes of late apoptotic cells and dead cells, will allow penetration of EB stain and binds with their fragments of concentrated DNA or apoptotic bodies, so it gives an orange-red fluorescence **[40]**. Thus, the morphological variations for untreated cells (control) and treated SKOV3 cells which were incubated with an IC_50_ value of SnO_2_ NPs and FA-SnO_2_ NPs for 24 h were studied using this test **[41]**. Briefly, in 6-well tissue plates, SKOV3 cells were grown overnight in the growth medium (2 ml). Then, after centrifugation, the supernatants from the tested plates were decanted and a fresh growth medium was supplemented which having the treated cells with SnO_2_ NPs and FA-SnO_2_ NPs. Afterward, the cells were washed with phosphate buffer saline (PBS) after 24 h of incubation and fixed in 4% formaldehyde for about 20 min. Lastly, using AO/EB (1 ml) the cells were stained for 2 min, and the nuclear morphology of all tested cells was observed via fluorescence microscopy (Carl Zeiss, Axiostar Plus, Germany) and the percentages of cell apoptosis were assessed.

#### 2.7.3 Detection of DNA damage using comet assay

The single-cell gel electrophoresis (comet assay) is a method used for the assessment of DNA damage in individual cells. The damaged DNA which has fragments and strand breaks will separate from intact cellular DNA under the effect of an electrophoretic field, resulted in a shape like a comet tail which can be observed using the fluorescence microscope. The comet assay includes many steps, which are described previously **[42,43]**, as follows: firstly, the frosted slides were prepared by covering the slides with 100 ml of agarose (low melting point, 1%) in PBS at 37°C and the agarose was frozen. Afterward, SnO_2_ NPs and FA-SnO_2_ NPs treated SKOV3 cells were centrifuged (2000 rpm for 5 min), and re-immersed in ice-cold PBS (step 1). Furthermore, about 10 ml of this prepared SKOV3 cell suspension was added to low melting point agarose (100 ml), and rapidly located on the frosted slide that was previously covered with low melting point agarose (step 2). Subsequently, these prepared agarose slides were incubated at 4°C for 2 h in cold lysis buffer for removing membranes and cellular material, resulting in separation of protein‐depleted nuclei containing supercoiled DNA (step 3). Next, the control and treated samples were dipped in high alkaline solution for disruption the hydrogen bonding between the DNA strands and also conversion alkali-labile lesions to DNA strand breaks (step 4). Then the slides were placed on horizontal gel electrophoresis with an electrophoresis buffer for 20 min (25 V, 300 mA, and pH 13.0) for construction of single-cell comets (step 5). Following, these slides were immersed in neutralization buffer (pH 7.5) three times for elimination of the extra alkali and detergents to ensure effective staining (step 6). Lastly, an ethidium bromide stain (20 μg/ml) was used for staining the slides and then observed with a fluorescence microscopy (step 7), such that every 100 images of random comets shape for each slide were captured using a computerized image analysis system which analyzed these images and estimated the comet parameters (step 8), using TriTek Comet Score™ software (TriTek Corp., Sumerduck, VA, USA). There are two common parameters called tail moment and tail DNA, which were intended for evaluating the obtained results. The tail moment parameter became to be known as Olive tail moment (OTM), where this parameter is assumed to be the finest parameter which was used for calculating the DNA damage based on DNA migration and DNA amount in the tail **[44]**, using the Eq (4):

OTM=tailmomentxtailDNA/100 (4)

### 2.8 Detection of apoptosis mechanisms

#### 2.8.1 Cell cycle investigation

Cell cycle assay was studied to detect the divisional phases of the cells, and to do this DNA content is utilized. The percentages of untreated SKOV3 cells and treated SKOV3 cells (treated for 24 h with an IC_50_ value of SnO_2_ NPs and FA-SnO_2_ NPs) through the different phases of the cell cycle were evaluated using a flow cytometry kit (Abcam, Propidium Iodide Flow Cytometry Kit, catalog ab139418) as previously mentioned **[45,46]**. Briefly, SKOV3 cells were seeded and incubated overnight then a cell density of about 1 x10^6^ cells were placed in a 6-well culture plate for 24 h. After that, these cells were treated with SnO_2_ NPs and FA-SnO_2_ NPs for 24 h. Subsequently, the cells were trypsinized, harvested, and overnight fixed in ice-cold ethanol (70%). Furthermore, the cells were centrifuged, washed with PBS twice, treated with RNase and then added to PI solution (40 μM ml-1 in PBS) for 30 min in the dark at 37°C for staining the control and treated cells. Finally, the percentages of cells existing in each cell cycle phase (sub-G1, G0/G1, S, and G2/M phases) were measured using DNA quantification using flow cytometry and the obtained results were displayed in the form of a histogram.

#### 2.8.2 Measurement of intracellular reactive oxygen species (ROS)

For detection of the potential mechanism of SnO_2_ NPs and FA-SnO_2_ NPs toxicity in human ovarian SKOV3 cancer cells, the concentrations of the intracellular generated ROS were measured using 2,7-dichlorofluorescin diacetate (DCFH-DA) assay as described before **[47,48]**. In brief, SKOV3 cancer cells were cultured in 12-well plates and incubated for 24 h. Then, the cells were treated with IC_50_ value of SnO_2_ NPs and FA—SnO_2_ NPs for 24 h. After that, the cells were detached using Trypsin EDTA, washed with PBS, and re-suspended in PBS having a DCFH-DA (10 Mm). Subsequently, the cells were incubated for 30 minutes at 37°C, where DCFH-DA can enter into the cells and reacts with ROS for the formation of fluorescent dichlorofluorescein (DCF). Afterward, the cells were lysed in alkaline solution, centrifuged and the supernatant was transferred to a microplate reader for measuring the concentration of fluorescent DCF at 485 nm excitation and 520 nm emission. Using the standard curve of hydrogen peroxide (measurement of the fluorescent intensity for different concentrations of hydrogen peroxide (μM)), we can estimate the concentration of ROS at different time interval through 24 h (6, 12, 18 and 24 h), in treated cells compared with the control untreated cells.

#### 2.8.3 Quantitative real-time PCR assay for genes expression studies

For detection of the mRNA expression levels of *Caspase-3*, *P53*, *Bcl-2*, and *Bax* as apoptotic related genes, the total cellular RNA was isolated from untreated (control cells) and the treated SKOV3 cells that had previously been treated with an IC_50_ value of SnO_2_ NPs and FA-SnO_2_ NPs for 24 h using RNA Purification Kit (ThermoFisher Scientific, catalog #K0731) consistent with the manufacturer’s mentioned instructions. Subsequently, the concentrations of the extracted RNA from all samples were measured by evaluating the ratio of A260/A280 using a spectrophotometer (ThermoFisher Scientific, NanoDrop 2000C) and the integrity of RNA was examined on agarose gel (1%) via gel documentation system (Universal Hood II, BioRad, Hercules, CA). Afterward, the first-strand cDNA was generated from the extracted RNA samples using a RevertAid First Strand cDNA Synthesis Kit (ThermoFisher Scientific, Catalog #K1621) according to the manufacturer’s instructions. Lastly, real-time PCR amplification was achieved using a Maxima SYBR Green qPCR kit (ThermoFisher Scientific, Catalog #K0221) on Applied Biosystems, StepOnePlus™ as a sequence detection system using specific primers (Invitrogen) for the studied apoptotic genes as listed in **Table 1**. Then, the relative quantification (RQ) of these genes were normalized to a b-actin gene as an internal housekeeping gene (control), compared with their expression levels in the untreated samples, and calculated using a comparative threshold cycle method (Ct), **[49].** All experiments were done in triplicate and expressed as a mean of three independent experiments.

**Table 1:** The primer sequences of the studied genes in qRT-PCR with GenBank accession numbers.

Sequences of the primers			
Gene	Forward primer	Reverse primer	GenBank accession number
***B-Actin***	F AGTTGCGTTACACCCTTTCTTC	R TCACCTTCACCGTTCCAGTTT	NM_001101.5
***Bcl-2***	F CATGTGTGTGGAGAGCGTCAA	R GCCGGTTCAGGTACTCAGTCA	NM_000633.3
***P53***	F CCCCTCCTGGCCCCTGTCATCTTC	R GCAGCGCCTCACAACCTCCGTCAT	NM_001126118.2
***Bax***	F GATCCAGGATCGAGCAGA	R AAGTAGAAGAGGGCAACCAC	NM_001291428.2
***Caspase-3***	F CAGAACTGGACTGTGGCATTGAG	R GGATGAACCAGGAGCCATCCT	NM_004346.4

**F:** Forward; **R:** Reverse.

#### 2.8.4 Evaluation of apoptosis-related proteins by Immunohistochemistry (IHC)

The immune-staining assessment for apoptosis-related proteins was carried out as adopted before **[50]**. For detection of the expression levels of Bax (Bcl-2-associated X), B-cell lymphoma-2 (Bcl-2), P53, and cleaved Caspase-3 (active Caspase-3), SKOV3 cells were cultured at a density of 2×10^5^ cells/well in 24-well plates and overnight incubated at 37°C. Then the cells were treated with an IC_50_ value of SnO_2_ NPs and FA-SnO_2_ NPs for 24 h. Furthermore, the treated and untreated cells were fixed in 4% formaldehyde (PFA) for 10 min, permeabilized in Triton-X-100 (0.5%) for 20 min, and blocked with hydrogen peroxide (3%) in methanol for 30 min for blocking the activity of endogenous peroxidase enzyme at room temperature. Subsequently, the expression levels of some apoptosis-related proteins were measured using specific antibodies for these proteins. So after blocking the untreated and treated cells, the cells were incubated overnight with anti-Bax (1:500; cat. no. ab53154; Abcam), anti-Bcl-2 (1:200; cat. no. ab59348; Abcam), Anti-p53 (1:100; cat. no. ab31333; Abcam) and anti-cleaved Caspase-3 (diluted 1:200; cat. no. ab2302; Abcam) as primary antibodies at 4°C. After that, they were washed with PBS and incubated with a horseradish peroxidase- conjugated goat anti-rabbit (1:1000; cat. no. ab214880; Abcam) as biotinylated secondary antibodies at room temperature for 1 h. Afterwards, the immune reactions were observed via incubation with developing diaminobenzidine solution (DAB) for 10 min and lightly counterstained with hematoxylin (Sigma-Aldrich, USA) for 3 min at room temperature. Finally, the stained cells were examined under a light microscope.

Semi-quantification immunostaining evaluation of the percent of positively stained cells (brownish granules in the nuclei for Caspase-3 and P53 and brownish granules in the cytoplasm for Bax and Bcl-2) was completed via counting about 1000 cells per tested slide in different power fields. The obtained results were expressed as an apoptotic index (AI) or labeling index (LI) using Eq **5**, which is considered to be a measure of apoptosis as previously mentioned **[51].**


Apoptoticindex(AI)=Numberofimmunelabelledpositivecells/Totalnumberofexaminedcells(1000)x100 (5)

#### 2.8.5 Western blotting analysis

Western blotting investigation was prepared as previously mentioned **[15,52],** for detection of the expressions levels of pro Caspase-3, cleaved Caspase-3 (active Caspase-3), P53, Bcl-2, and Bax apoptotic proteins. In brief, SKOV3 cells were cultured in 6-well plates at a density of 3×10^5^ cells/well for overnight incubation and then treated with the IC_50_ value of SnO_2_ NPs and FA-SnO_2_ NPs for 24 h at 37°C. After washing the cells with ice-cold PBS, the treated and untreated cells were centrifuged and incubated with protease inhibitor and lysis buffer for solubilization and extraction of the total proteins. Afterward, the concentrations of the extracted proteins were quantitated with bicinchoninic acid protein (BCA) test. Subsequently, the proteins were denatured by adding SDS-PAGE loading buffer (Tris-HCl (pH 6.8), SDS, DTT, bromophenol blue, and glycerol), and boiling for 10 min. Afterward, the proteins (30 μg) were separated by size via sodium dodecyl sulfate-polyacrylamide gel (SDS-PAGE) electrophoresis in Tris-glycine electrophoresis buffer using acrylamide gels (12%). After electrophoresis, the separated proteins were transferred to Polyvinylidene difluoride (PVDF) membranes in transferring buffer (methanol, Tris -HCl, and glycine). Then, after soaking the membranes in blocking buffer (skimmed milk), they were incubated with specific primary antibodies for Bax (ab53154; Abcam), Bcl-2 (ab59348; Abcam), p53 (ab31333; Abcam) and proCaspase-3 (ab32150; Abcam), cleaved Caspase-3 (ab2302; Abcam) and GAPDH (ab8245; Abcam), for overnight incubation at 4˚C. GAPDH was used as a loading control. After washing with TBS-0.05% Tween 20 (TBST) solution, the membranes were incubated with horseradish peroxidase -conjugated goat anti-rabbit secondary antibodies (ab6721; Abcam) at 2 h at room temperature. Subsequently, the formed bands or blots were visualized using x-ray photographic instrument. Finally, the resulted apoptotic protein levels were evaluated using densitometric analysis via AlphaEase TM FC StandAlone V.4.0.0 software and expressed with a fold change comparing to the control cells.

### 2.9 Evaluation of the toxicity of the prepared FA- SnO_2_ NPs by in vivo studies

The prepared FA-SnO_2_ NPs toxicity was investigated in vivo against healthy tissues. Briefly, healthy adult Wister rats weighing, 200–250 g were used as the experimental model. The Rats were obtained from the National Research Center (animal house), Egypt. Then, the rats were left for 14 days for acclimatization before the testing procedure. The tested Rats were housed and kept under standard environmental conditions (Temperature at 23± 2°C, humidity 20.37%, and 12-hour light/dark cycle). All the experimental rats were handled in agreement with the Institutional Animal Care and Use Committee (Animal ethics approval number: CU/I/F/15/21) of the Faculty of Science, Cairo University, Egypt. The tested rats were distributed randomly in four separate groups, each containing eight rats. The first group (A) was intraperitoneally treated with normal saline (control), while the second, third and fourth group were intraperitoneally treated with different doses of FA-SnO_2_ NPs as follow: 2nd group treated with 50 mg/kg (B); 3rd group treated with 100 mg/kg (C); and 4th group treated with 200 mg/kg (D) for 14 successive days.

#### 2.9.1 Hematological parameters analysis

At the end of experimental time (14 days), the rats of all tested groups were anesthetized by ketamine and xylazine, then the blood samples from the abdominal aorta of rats were collected within sterile Wassermann tubes with an anticoagulant (tripotassium EDTA), for examination the changes in hematological parameters (white blood cell (WBC) count, red blood cell (RBC) count, hemoglobin (Hb) concentration, hematocrits (HCT), mean corpuscular volume (MCV), platelet (PLT) count, and RBC distribution width (RDW)) using RT-7600 Auto Hematology Analyzer (Rayto Life and Analytical Sciences Co., Ltd, China).

#### 2.9.2 Biochemical parameters analysis

For biochemical analysis, we collected the blood on non-anticoagulant Wassermann tubes, then the serum was collected in Eppendorf’s tubes after centrifugation at 3000 rpm for 15 minutes using for measurement the levels of different parameters (alkaline phosphatase (ALP), alanine aminotransferase (ALT), aspartate aminotransferase (AST), total lipids (TLs), urea, creatinine (Cr), uric acid and total protein (TP). ALP, ALT, and AST activities were determined according to the methods described before **[53,54]** and Isoenzyme of Creatine kinase (CK-MB) activity was evaluated according to the methods described before **[55]**. Furthermore, the concentrations of TLs, urea, uric acid, creatinine, and TP were determined according to the methods described previously **[56–60].**

#### 2.9.3 Histopathological investigations

For investigation histopathologic changes, the rats of all tested groups were euthanized, and then their tissues of liver, kidney, lung, and brain were removed and fixed immediately in formalin (10%) for routine paraffin inserting. Using the rotary microtome, Paraffin sections blocks of the selected tissues were cut and put on glass slides. Then, the prepared sections were stained using hematoxylin-eosin (H–E) method **[61,62]**. Lastly, the stained sections were investigated using a light microscope where the photomicrographs of the tissue samples were recorded.

#### 2.10 Statistical analysis

All tests were carried out in triplicates and the obtained data were stated as mean ± SD. The differences between means were evaluated using one-way ANOVA. All statistical evaluates were achieved via SPSS 17.0 software. Levels of p ≤ 0.05, p ≤ 0.01, and p ≤ 0.001 were taken as statistically significant.

## 3. Results

### 3.1 Physicochemical characterization

#### 3.1.1 Particles size analysis

**Fig 1:**
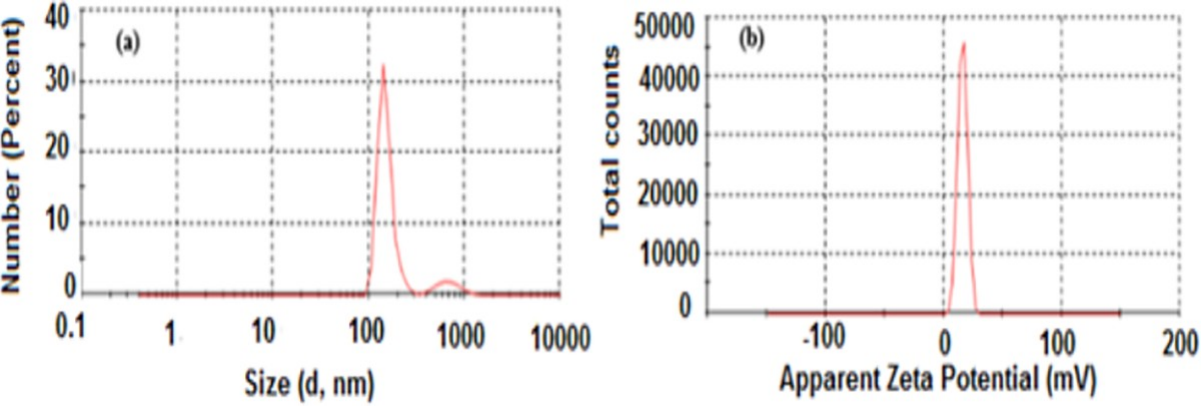
The average particle size (a) and Zeta potential value (b) of the prepared SnO_2_ NPs using DLS technique.

**Fig 2:**
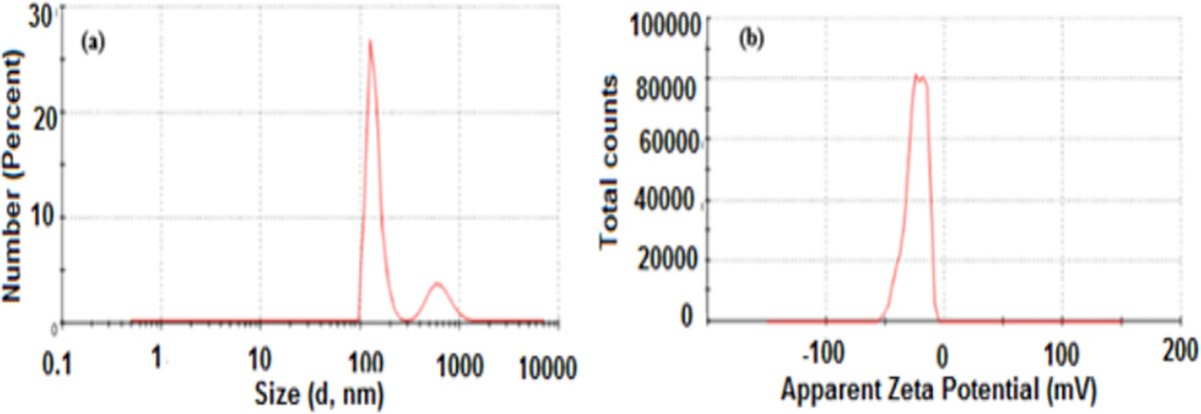
The average particle size (a) and Zeta potential value (b) of the prepared FA-SnO_2_ NPs using DLS technique.

The surface charge and particle size distribution of SnO_2_ NPs and FA-SnO_2_ NPs were measured using the dynamic light scattering (DLS) technique and the results are displayed in **Figs 1** and **2**, respectively. As shown in these figures, both SnO_2_ NPs (**Fig 1A**) and FA-SnO_2_ NPs (**Fig 2A**) exhibited a narrow distribution interval with average particles size of about 151 and 158 nm, respectively. Zeta potential of the prepared SnO_2_ NPs was found to be 15.8 mV **(Fig 1B)** and the Zeta potential of SnO_2_ NPs after conjugation with folic acid was measured as -24 mV **(Fig 2B)**.

#### 3.1.2 UV-vis and FT-IR measurements

**Fig 3:**
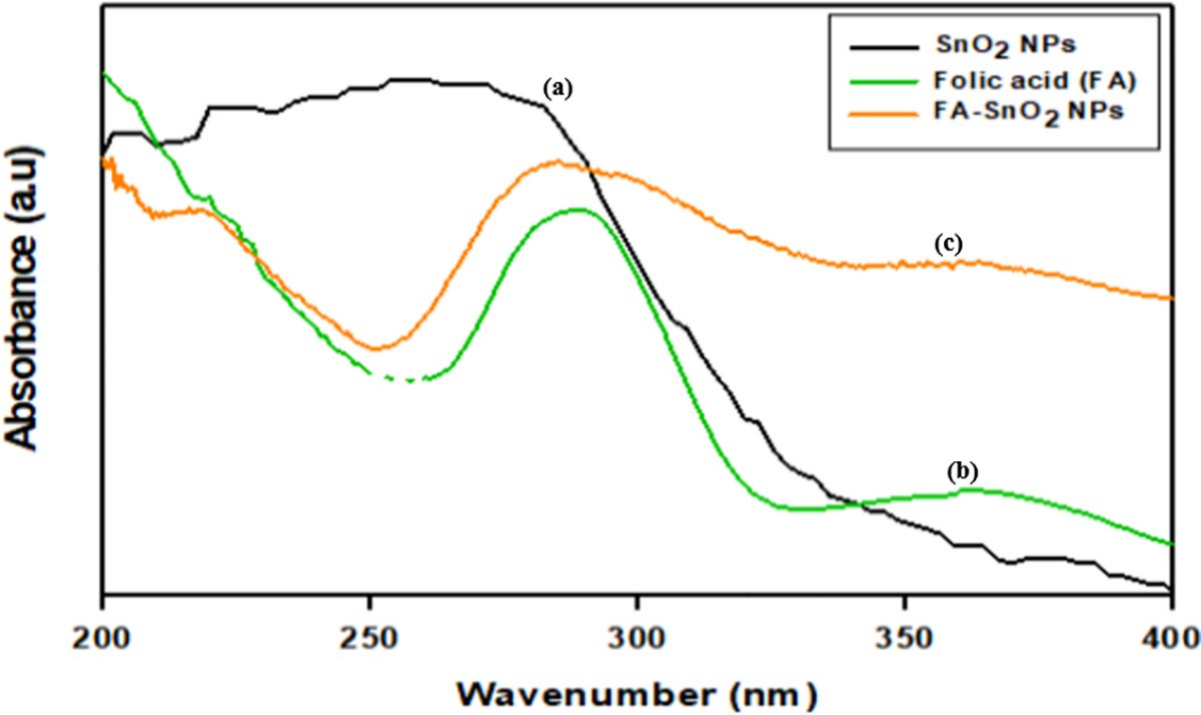
UV absorption spectra of the prepared SnO_2_ NPs (a), Folic acid (b) and FA-SnO_2_ NPs (c).

The successful preparation of the SnO_2_ NPs and coated SnO_2_ NPs with folic acid were confirmed using UV-Vis measurements. The UV-visible spectra of folic acid, SnO_2_ NPs, and FA-SnO_2_ NPs in methanol are presented in **Fig 3**. As can be seen in **Fig 3A**, SnO_2_ NPs spectrum showed an absorption edge at 231.5 nm. In the spectrum of FA **(Fig 3B)**, two absorption bands, around 289 nm and 360.5 nm were observed. The spectrum of FA-SnO_2_ NPs (**Fig 3C**), showed an appearance of three bands at 219 nm, 284 nm, and 362 nm.

**Fig 4:**
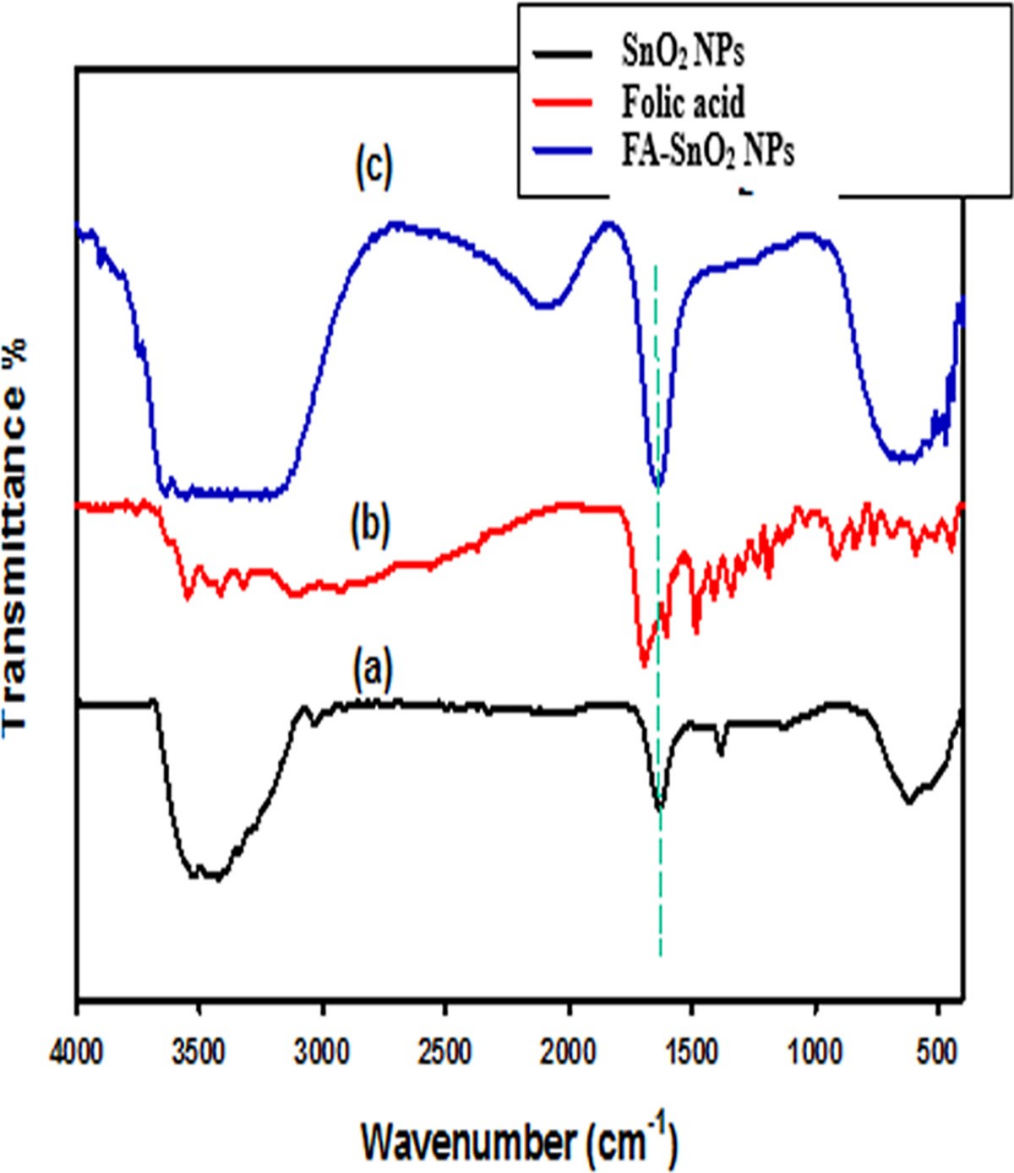
FTIR spectrum of the (a) SnO_2_ NPs, (b) Folic acid (FA), and (c) FA-SnO_2_ NPs.

The conjugation of SnO_2_ NPs with FA was also confirmed via FT-IR measurements. According to the FT-IR spectrum of SnO_2_ NPs (**Fig 4A)**, a wide peak was observed at around 3526 cm^-1^ that is assigned to–OH stretching of methanol and the water molecules that absorbed on the SnO_2_ NPs surface. Also, the presence of methanol is confirmed by C–H stretching in the range 3456–3032 cm^-1^ and bending at a value of about 1381 cm^-1^. The peak at 1126 cm^-1^ is related to Sn–O asymmetric. Furthermore, the appearance of the O-Sn-O bond was detected at 617 cm^-1^, suggesting that the obtained SnO_2_ NPs were crystallized. In the FT-IR spectrum of free folic acid **(Fig 4B)**, the–NH,–OH, and -CH were characterized by the peaks that appeared at 2630, 2839, 2924, 3109, 3325, 3417, and 3456 cm^-1^. Moreover, there was a strong peak at 1697 cm^-1^ which related to carboxylic acid groups. Furthermore, the peaks related to C–O stretching were observed at 1296 cm^-1^. The peak related to C = C stretching was detected at 1604 cm^−1^. In the spectrum of FA-SnO_2_ NPs **(Fig 4C)**, the shift of the O-Sn-O peak from 617 to 471 cm^-1^ compared to the O-Sn-O of the uncoated SnO_2_ NPs confirm the conjugation of FA with the surface of SnO_2_ NPs. Furthermore, the appearance of a broad band centered at 3410 cm^–1^ due to the O-H stretching of–OH of FA may support the incorporation of FA into SnO_2_ NPs.

#### 3.1.3 XRD analysis

**Fig 5:**
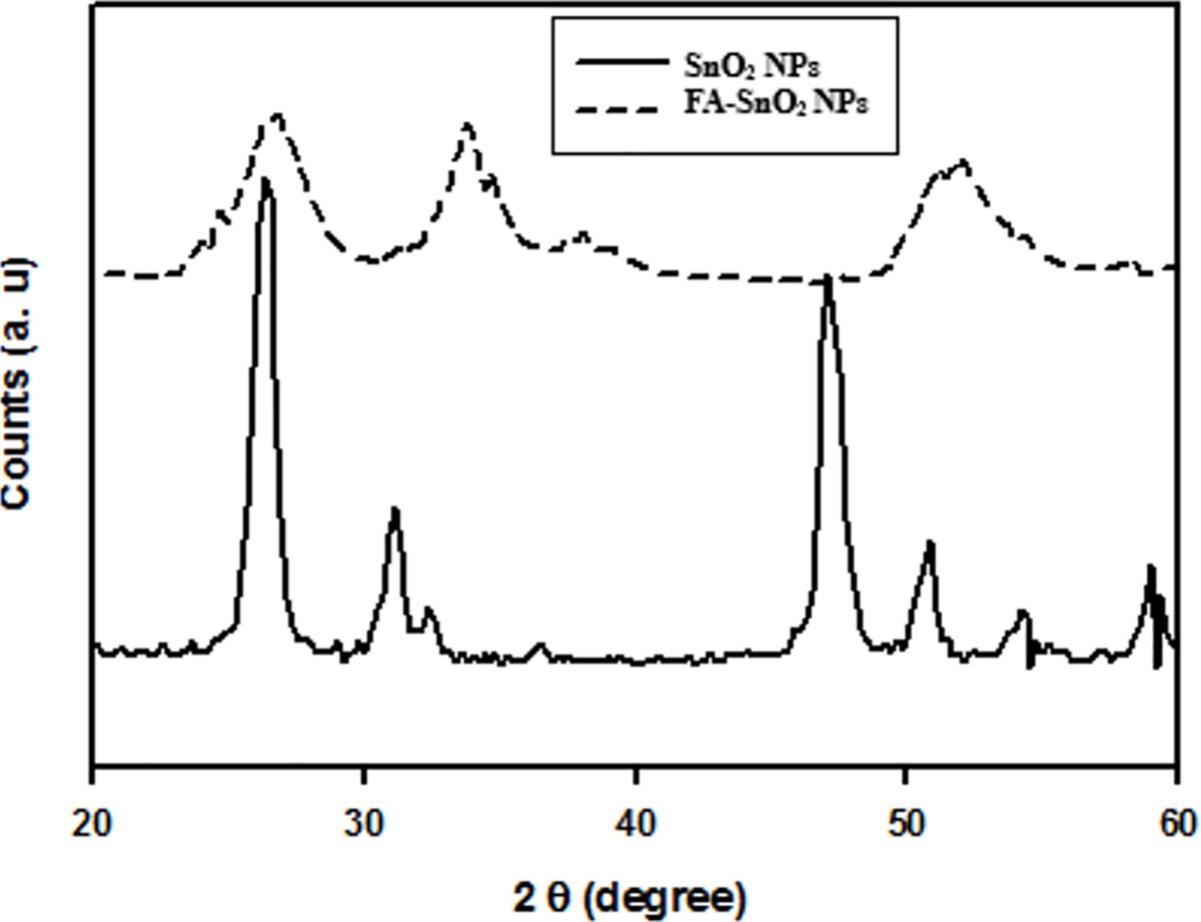
The X-ray diffraction pattern of the prepared SnO_2_ NPs and FA-SnO_2_ NPs.

**Fig 5** represented the XRD patterns of SnO_2_ NPs and coated SnO_2_ NPs with folic acid (FA-SnO_2_ NPs) samples. The peaks with 2θ of characteristics of SnO_2_ NPs values at about 26.5°, 34.2°, 37.9°, 51.7°, 54.4°, and 57.7° are ascribed to the (110), (101), (200), (211), (220) and (002), planes, respectively, indicating the formation of SnO_2_ NPs with a tetragonal structure in the rutile phase. For FA-SnO_2_ NPs sample, diffraction peaks broadened and shifted to higher values, suggesting some amorphous character.

The average grain sizes of uncoated and coated SnO_2_ NPs were also estimated by Scherrer’s equation **[63,64]**:

$$D=\frac{K\lambda }{\beta cos\theta }$$ (6)

where, D is the average grain size, K the shape factor (taken as 0.94 for a spherical shape), λ is the X-ray wavelength, β is the full width at half maximum (FWHM) intensity and θ is the Bragg angle.

The calculated average particle size of SnO_2_ NPs is around 101 nm and decreases to 54 nm after FA coating.

#### 3.1.4 Scanning electron microscope (SEM)

**Fig 6:**
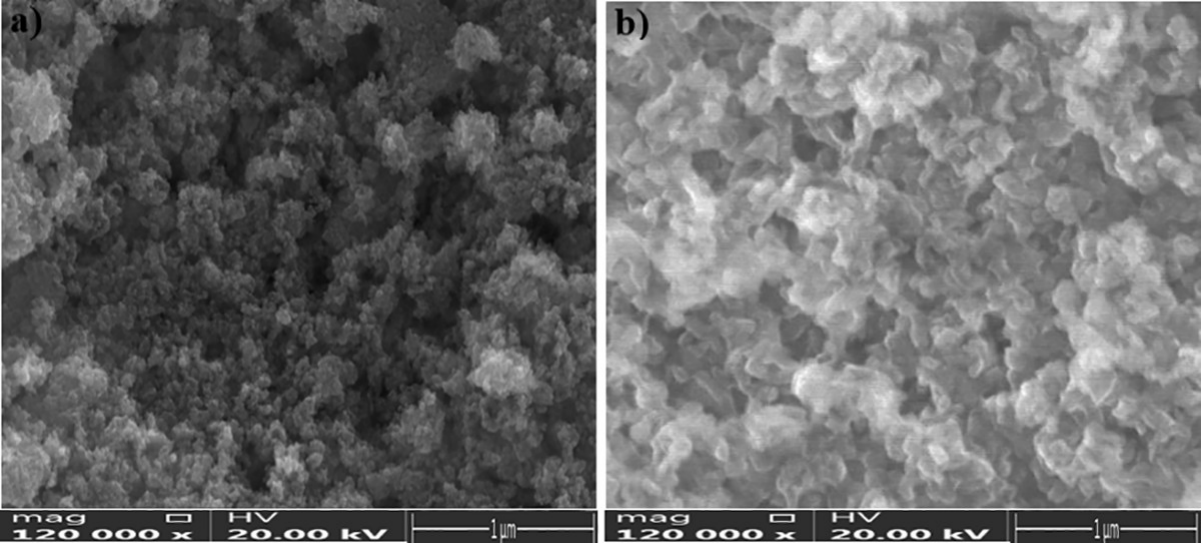
SEM image of the prepared SnO_2_ NPs (a) and FA-SnO_2_ NPs (b).

SEM analysis was performed to investigate the surface morphological of SnO_2_ NPs and FA-SnO_2_ NPs. As showed in **Fig 6**, SEM image of FA-SnO_2_ NPs (**Fig 6B**) revealed that folic acid didn’t affect spherical homogeneous particles of the SnO_2_ NPs surface (**Fig 6A**) in well distributed manner over a scanned area.

#### 3.1.5 Transmission electron microscope (TEM)

**Fig 7:**
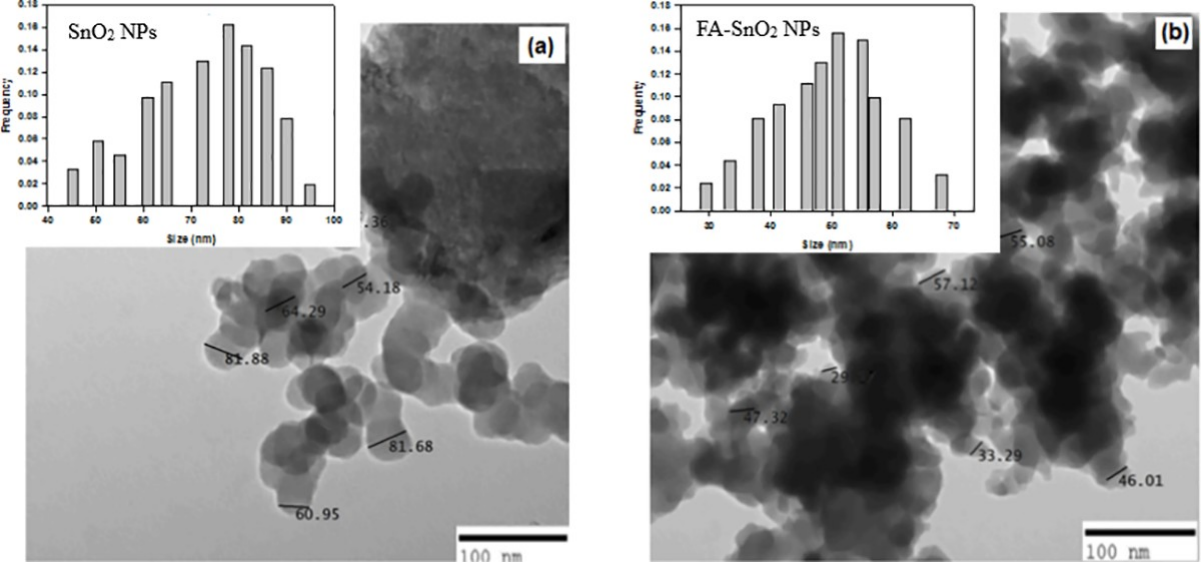
TEM image of the prepared SnO_2_ NPs (a) and FA-SnO_2_ NPs (b).

The morphology and particle size of the SnO_2_ NPs and FA-SnO_2_ NPs were further investigated by TEM (**Fig 7**). It was obvious that both the SnO_2_ NPs and FA-SnO_2_ NPs exhibited almost spherical morphology and their size distribution indicated that the average size of SnO_2_ NPs and FA-SnO_2_ NPs were about 74 and 49 nm, respectively.

### 3.2 Effect of SnO_2_ NPs and FA-SnO_2_ NPs on cells viability and morphology

**Fig 8:**
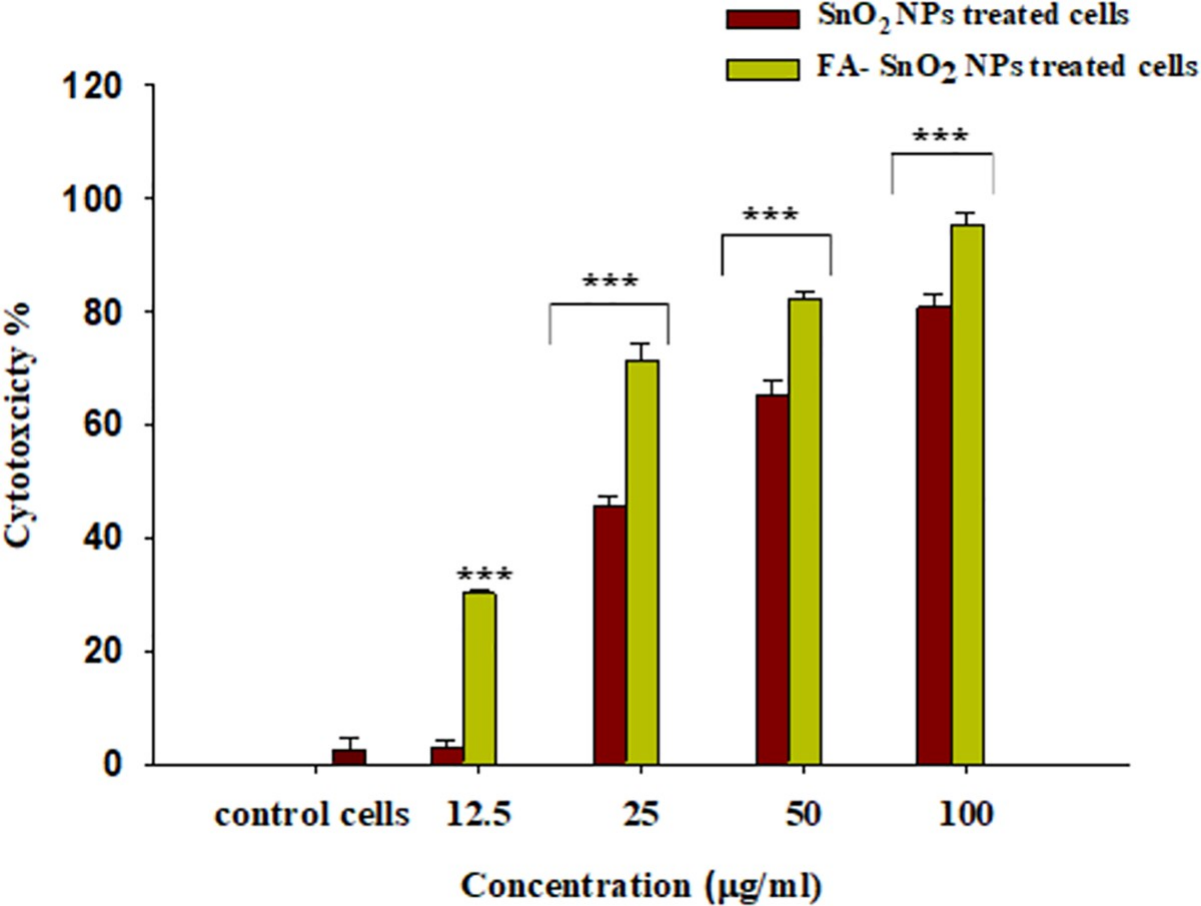
The relationship between the cell cytotoxicity (%) of SKOV3 cells treated with different concentrations of SnO_2_ NPs and FA-SnO_2_ NPs compared with control group, the values displayed as mean ± SD of three independent tests; ***P < 0.001.

**Fig 9:**
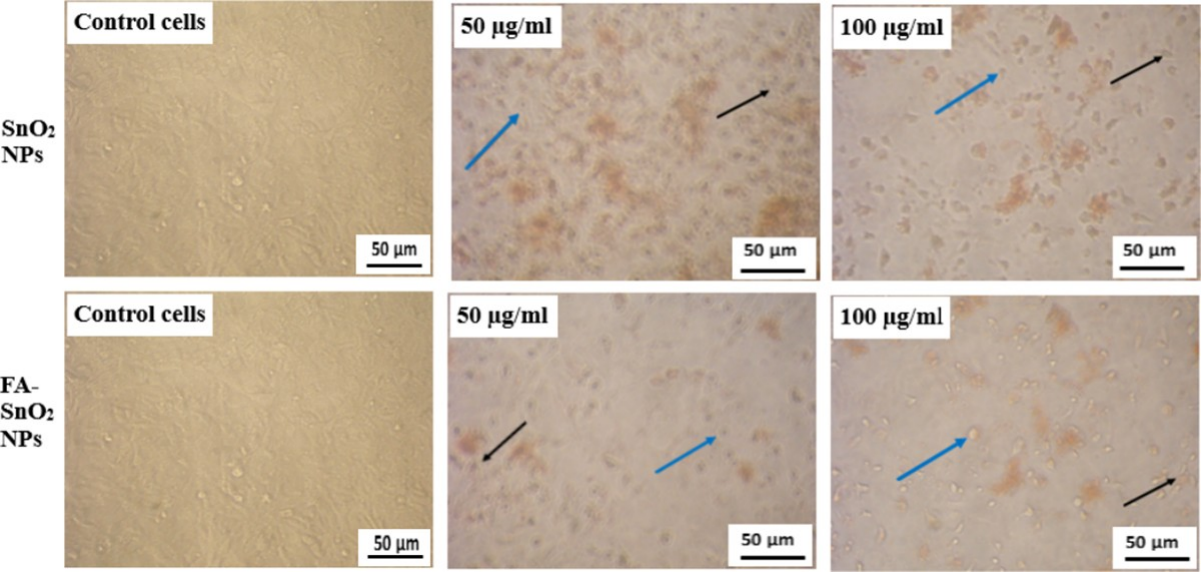
Microscopic observation of the morphological changes in SKOV3 cancer cell lines after treatment with different concentrations of SnO_2_ NPs and FA-SnO_2_ NPs through 24 h incubation as compared with normal control cells. Blue arrow indicates rounded cells and black arrow indicates apoptotic bodies.

The efficacy of SnO_2_ NPs and FA-SnO_2_ NPs on the growth and morphology of SKOV3 cells using MTT assay is shown in **Figs 8 and 9**, respectively. Comparing with control group (**Fig 8**), the % of cytotoxicity was significantly increased in FA-SnO_2_ NPs and SnO_2_ NPs treated SKOV3 cells with increasing concentration of SnO_2_ NPs and FA-SnO_2_ NPs (12.5, 25.5, and 100 μg/ml) for 24 h incubation (P < 0.001). Moreover, FA-SnO_2_ NPs have shown stronger cytotoxic effects than SnO_2_ NPs against SKOV3 cells at each tested concentration, where at the highest tested concentration (100 μg/ml), the % of cytotoxicity of SnO_2_ NPs and FA-SnO_2_ NPs was found to be 80.76 ± 2.4% and 95.32 ± 2.3%, whereas at the lowest concentration (12.5 μg/ml), were found to be 2.74 ± 1.5% and 30.2 ± 0.48%, respectively. Also, using dose response curves, the calculated IC50 values for the SnO_2_ NPs and FA-SnO_2_ NPs indicated that FA-SnO_2_ NPs had the strongest anti-proliferative effect against SKOV3 cancer cells with lower IC50 value (14.2 ± 0.76 μg/ml) compared to SnO_2_ NPs (IC50 = 47.87 ± 0.68 μg/ml) for 24 h incubation.

Comparison of the cytotoxic effect of SnO_2_ NPs and FA-SnO_2_ NPs on the morphology of SKOV3 cells with that of untreated one (**Fig 9**) showed the cells treated with FA-SnO_2_ NPs exhibited more morphological damage of apoptotic cell (phase-dense nucleus, rounded cells and apoptotic bodies) than SnO_2_ NPs treated cells compared to control cells (strongly adherent cells to the surface and typically epithelial monolayer). This result is in line with the above-gotten cytotoxicity results.

For additional confirmation the toxic effect of the SnO_2_ NPs and FA-SnO_2_ NPs on the viability of SKOV3 cells, the activity of LDH enzyme was measured externally. This test is used as an indicator for cell cytotoxicity by assessment of the permeability of cell membrane. The results indicated that there was a highly significant increase in leakage percentage of LDH in FA-SnO_2_ NPs (92.5 ± 1.8), and SnO_2_ NPs (79.7 ± 1.2) treated SKOV3 cells, as compared with the percentage of leakage in control cells (12.8 ± 0.5), P < 0.001. In addition, there was a higher significant increase of leakage percentage of LDH in FA-SnO_2_ NPs treated cells than SnO_2_ NPs treated cells, P < 0.001.

### 3.3 Flow cytometry analysis

**Fig 10:**
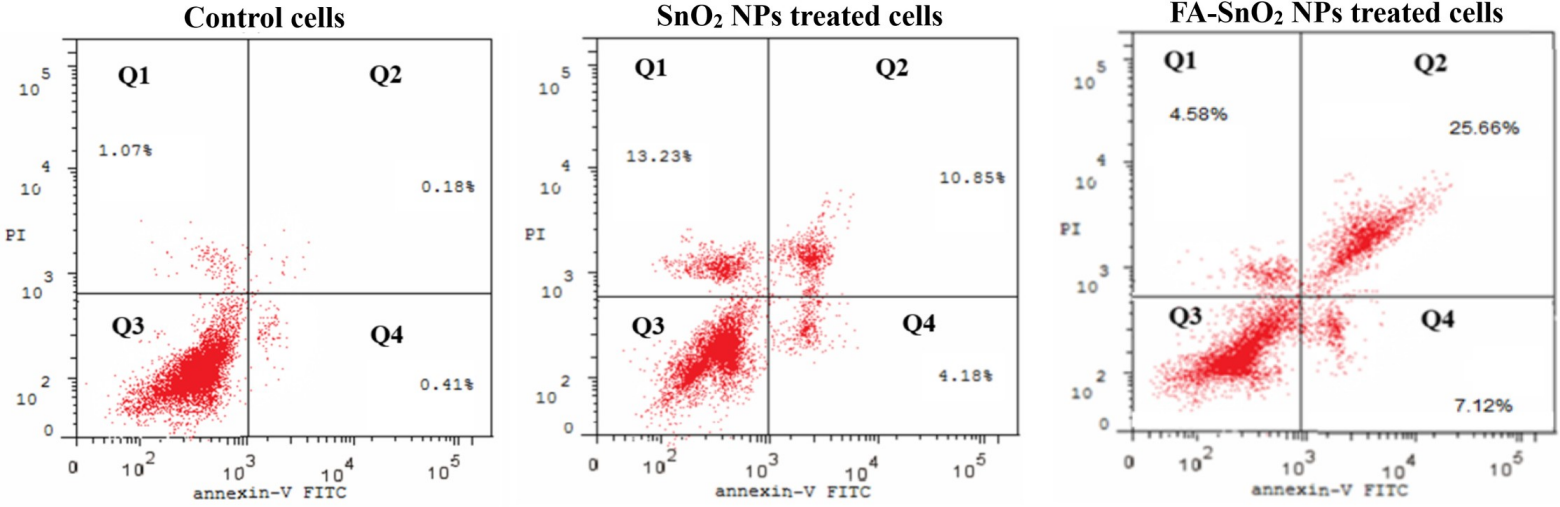
Apoptotic profile of SKOV3 cells using flow cytometry analysis, displaying viable cells percentages: Q3 (An^-^, PI^-^), early apoptotic cells: Q4 (An^+^, PI^-^), late apoptotic cells: Q2 (An^+^, PI^+^) and necrotic cells: Q1 (An^-^, PI^+^), for control cells, SnO_2_ NPs treated cells, and FA-SnO_2_ NPs treated cells.

The apoptosis potential of SnO_2_ NPs and FA-SnO_2_ NPs against SKOV3 cells was investigated using Annexin V-FITC/PI staining. The obtained data produced by flow cytometry were plotted in two dot plots (PI is displayed against Annexin V- FITC), where the plots were divided in four different regions corresponding to Q1 (necrotic cells; PI positive and Annexin negative), Q2 (late apoptotic cells; PI positive and Annexin positive), Q3 (viable cells; PI negative and Annexin negative) and Q4 (early apoptotic cells, PI negative and Annexin positive). As seen in **Fig 10,** there was an increase of significant percentages of early apoptotic, late apoptotic, and necrotic cells in FA-SnO_2_ NPs, and SnO_2_ NPs treated SKOV3 cells, as a compared to the control cells, P < 0.001. Besides, FA-SnO_2_ NPs induced a higher percentage of apoptotic cells than SnO_2_ NPs in treated SKOV3 cells, P < 0.001.

### 3.4 AO/EB staining assay

**Fig 11:**
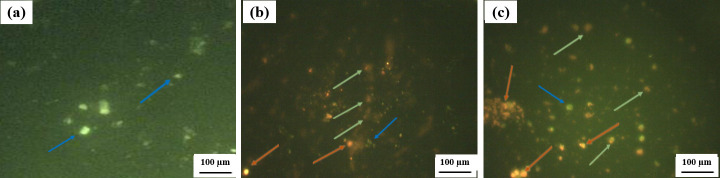
Fluorescence microscope investigation for the nuclear morphological alterations in control cells (a), SnO_2_ NPs treated cells (b), and FA-SnO_2_ NPs treated cells (c) using AO/EB staining. Blue arrow indicates viable cells, green arrow indicates early apoptotic cells and orange arrow indicates late apoptotic cells.

AO/EB dual staining is known as a qualitative and quantitative method for identifying apoptosis, so the nuclear morphology alterations and percentages of apoptotic cells that associated with apoptosis induction in SKOV3 cancer cells under the effect of both SnO_2_ NPs and FA-SnO_2_ NPs were confirmed using AO/EB technique and the results are displayed in **Fig 11A–11C**. A green AO, yellow-green AO, and orange EB nuclear staining were detected for viable, early, and late apoptotic cells, respectively in SnO_2_ NPs **(Fig 11B)** and FA-SnO_2_ NPs **(Fig 11C)** treated cells as compared to control cells **(Fig 11A)**. Furthermore, there was a highly significant percentage of apoptotic cells in FA-SnO_2_ NPs (90 ± 1.8) and SnO_2_ NPs (66 ± 0.88) treated cells as compared to control cells (8 ± 1.2), P < 0.001. In addition, FA-SnO_2_ NPs treated cells induced stronger toxic effects on SKOV3 cells than treated SnO_2_ NPs, P < 0.001.

### 3.5 Comet assay

**Fig 12:**
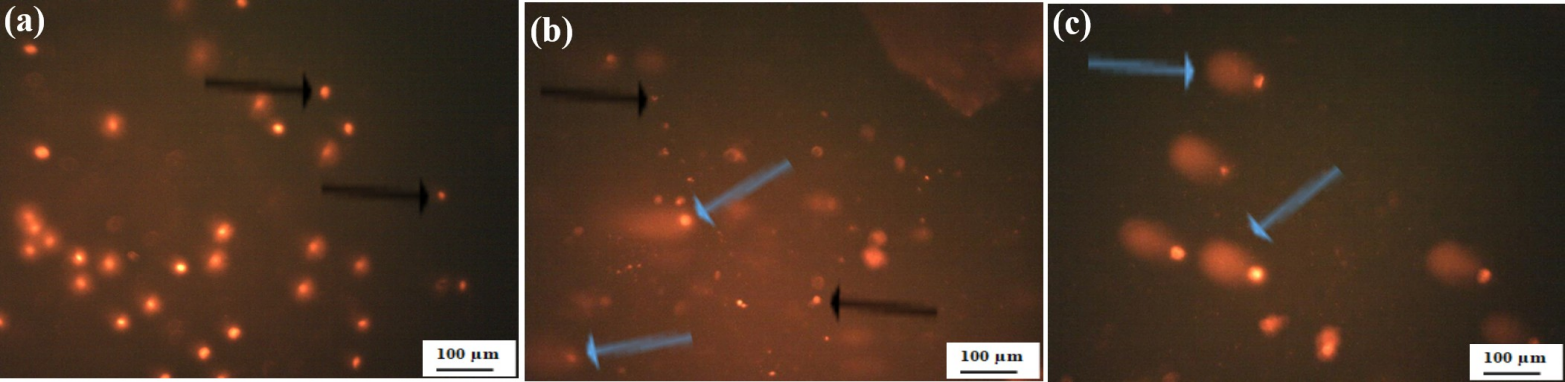
Fluorescence microscopic pictures of comet nucleus in SnO_2_ NPs treated cells (b), and FA- SnO_2_ NPs treated cells (c) compared with intact nucleus in control cells (a). Black arrow indicates intact nucleus of viable cells and blue arrow indicates comet nucleus of apoptotic cells.

The single-cell gel electrophoresis (comet assay) is used as a marker of DNA damage owing to apoptosis induction in SKOV3 treated cells. As seen in **Table 2**, there is a significant increase in OTM in treated cells as compared to control cells (P < 0.001). In addition, there was a higher significant increase of OTM in FA-SnO_2_ NPs treated cells than SnO_2_ NPs treated cells (P < 0.01). Furthermore, the investigated fluorescence microscopic pictures (**Fig 12A–12C**), revealed that the control cells had an intact nucleus (**Fig 12A**), whereas in case of SnO_2_ NPs and FA-SnO_2_ NPs treated cells, a comet like formation is visible, which indicate single/double stranded breakage. However, increasing the tail length of the FA-SnO_2_ NPs (**Fig 12C**) treated cells compared to SnO_2_ NPs (**Fig 12B**) treated cells is taken as an indication of the reduction of nuclear intensity resulted from a high accumulation of DNA in the tail.

**Table 2:** The parameters of the comet assay for measurement of the DNA damage in SnO_2_ NPs and FA-SnO_2_ NPs treated SKOV3 cells compared with control cells.

The tested cells	Tail Length (px)	%DNA in Tail	Tail Moment	Olive tail moment
**Control untreated SKOV3 cells**	10.25 ± 1.5	2.91 ± 0.5	0.213726 ± 0.08	0.42 ± 0.02
**SnO**_**2**_ **NPs treated SKOV3 cells**	7.67± 1.8	7.39 ± 1.3**	0.939902 ± 0.1**	1.5 ± 0.05***
**FA-SnO**_**2**_ **NPs treated SKOV3 cells**	11.87 ± 2.2	9.73 ± 2.3**	1.442354 ± 0.09***	2.5 ± 0.2***

Data displayed as mean ± SD of three independent tests

**P < 0.01 and ***P < 0.001.

### 3.6 Cell cycle examination

**Fig 13:**
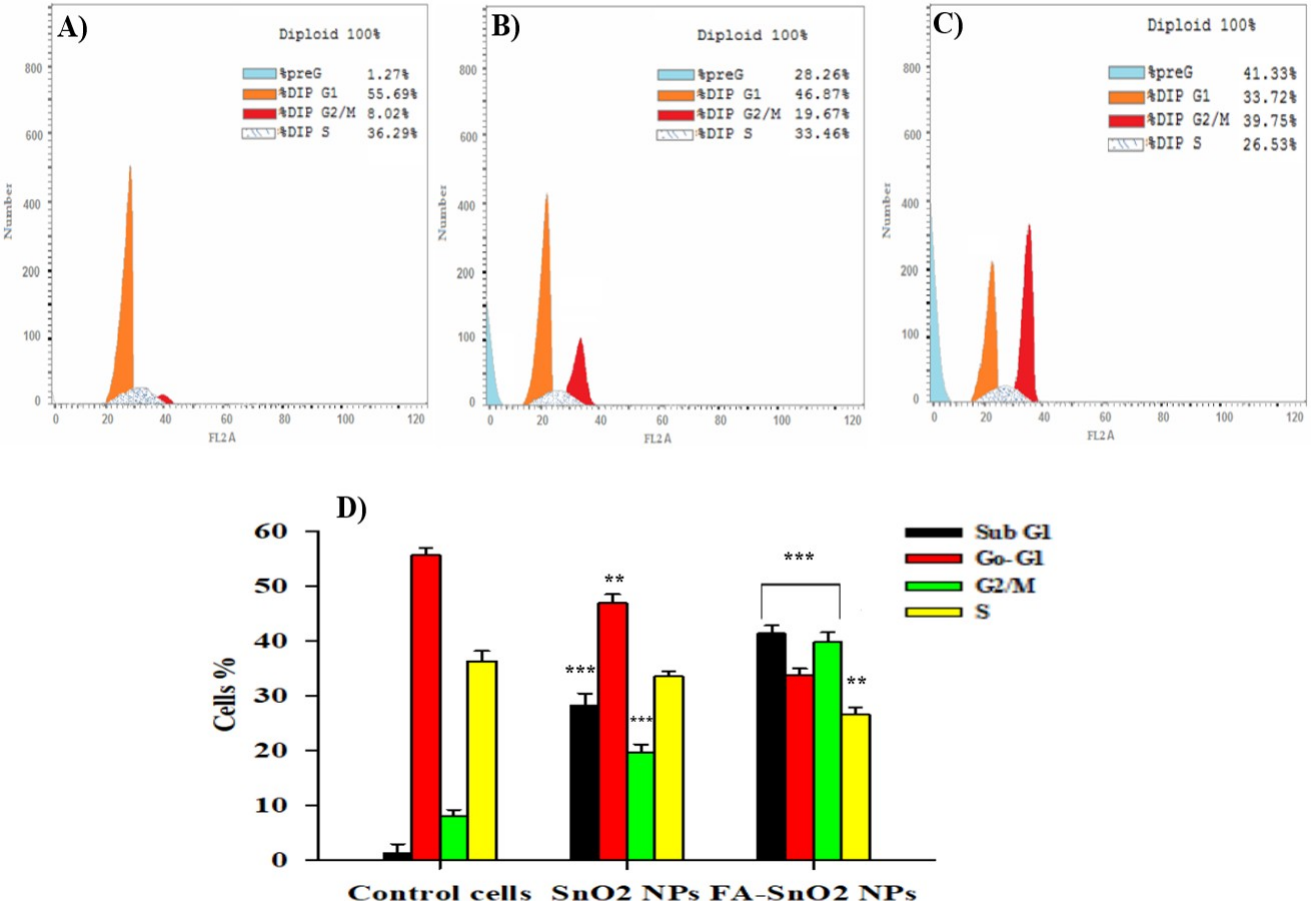
Flow cytometry analysis of cell cycle distribution in different phases in control cells (A), SnO_2_ NPs treated cells (B) and FA-SnO_2_ NPs treated cells (C). A representative histogram (D), showing the percentages of the cells in each phase of the cell cycle in SnO_2_ NPs and FA—SnO_2_ NPs treated cells comparing with control cells. Data represented as mean ± SD of three independent tests; **P < 0.01 and ***P < 0.001.

The influence of SnO_2_ NPs and FA-SnO_2_ NPs on SKOV3 cell cycle distribution was studied using flow cytometry. As seen in **Fig 13A–13D**, the control cells (**Fig 13A**), did not exhibit any peaks related to existing of apoptotic cells in the sub- G1 phase, whereas FA-SnO_2_ NPs (**Fig 13C**), and SnO_2_ NPs (**Fig 13B**), induced a higher significant percentage of apoptotic cells in the sub-G1 and G2/M phases of SKOV3 treated cells comparing with control cells, P < 0.001. Furthermore, there was a significant increase of cell population percentage of sub- G1 and G2/M phases in FA-SnO_2_ NPs treated cells than SnO_2_ NPs treated cells, P < 0.001. In addition, the percentage of cell population in the G0/G1 and S phases of FA-SnO2 NPs and SnO2 NPs treated cells was lower than the percentage in control cells. A typical histogram displayed the percentages of cell population in each cell cycle phase related to SnO_2_ NPs and FA-SnO_2_ NPs treated cells comparing with control cells is shown in **Fig 13D**.

### 3.7 Toxicity of metal oxide NPs mediated reactive oxygen species (ROS)

**Fig 14:**
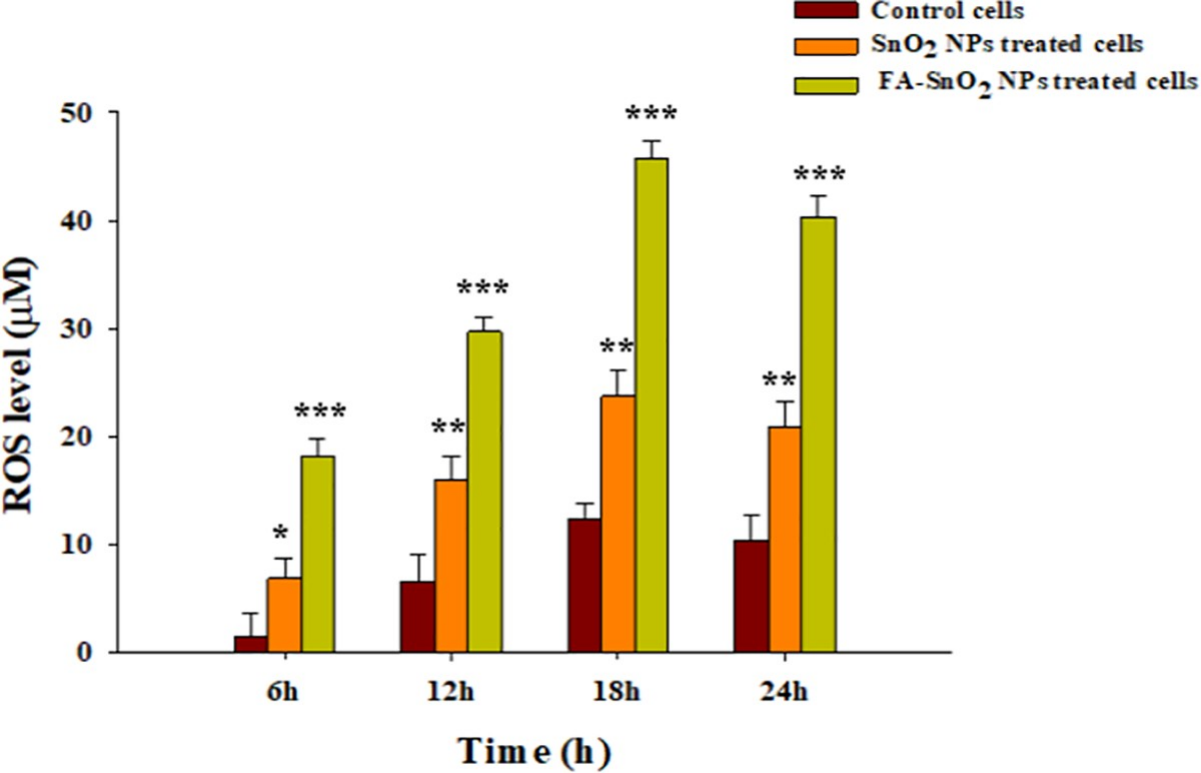
The intracellular reactive oxygen species concentration (ROS) in SnO_2_ NPs and FA-SnO_2_ NPs treated SKOV3 cells at different time intervals of treatment (6, 12, 18 and 24 h). All values displayed as mean ± SD of three independent tests; *P < 0.05, **P < 0.01 and ***P < 0.001.

To determine the potential mechanisms through which SnO_2_ NPs and FA-SnO_2_ NPs induce toxicity toward SKOV3 cancer cells, the markers of oxidative stress such as ROS were determined in control and treated cells through different time intervals for 24 h incubation by measuring the oxidation of DCFH-DA (non-fluorescent) for its highly fluorescent DCF, where DCFH-DA will react with hydrogen peroxide (that is well characterized ROS) and emits fluorescent DCF. As seen in **Fig 14**, with increasing the incubation time (6–18 h), the concentration of ROS increased in FA-SnO_2_ NPs treated cells than SnO_2_ NPs treated cells compared with control group, P < 0.001, reached a maximum value at 18 h incubation.

### 3.8. Genes expression, Immunohistochemistry, and Western blotting analyses

**Fig 15:**
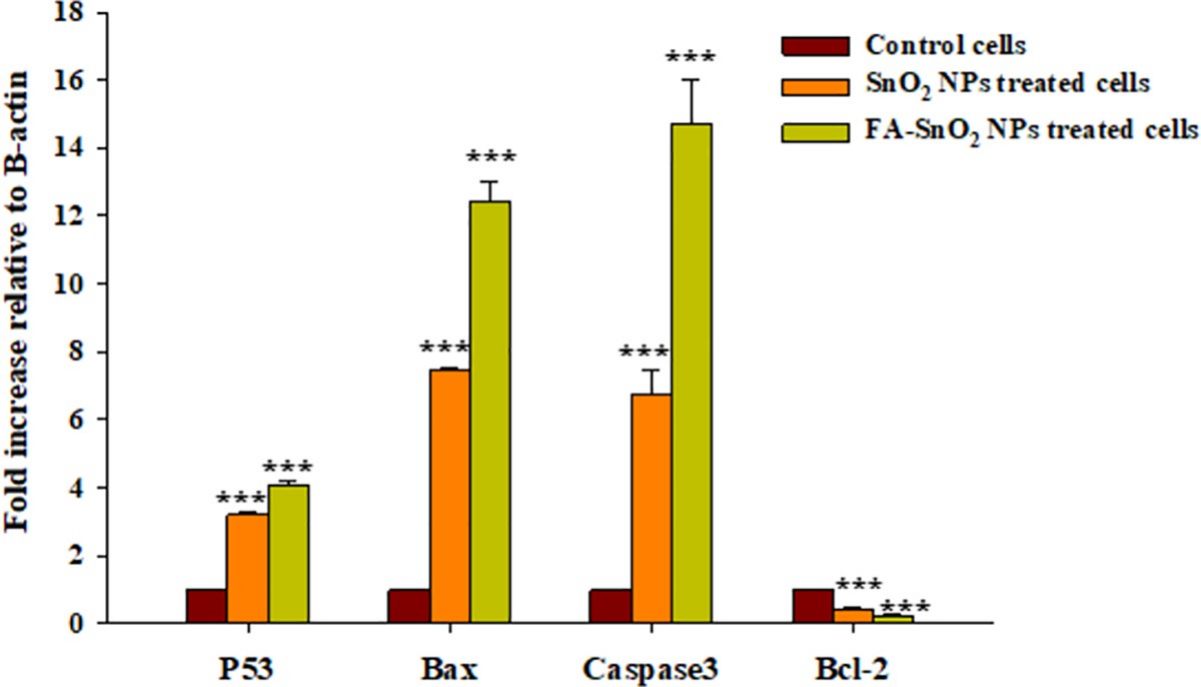
Effect of SnO_2_ NPs and FA-SnO_2_ NPs on the expression levels of *P53*, *Bax*, *Caspase-3*, and *Bcl-2* in SKOV3 treated cancer cells compared to control cells, using RT-PCR. Data represented as mean ± SD of three independent tests; *** P < 0.001.

For detection of the possible pathways for induction SKOV3 cells apoptosis, the expression levels of mRNA of some apoptotic genes coding for apoptosis related proteins (*P53*, *Bax*, *Caspase-3*, and *Bcl-2*), were analyzed using quantitative real-time PCR in SnO_2_ NPs and FA-SnO_2_ NPs treated SKOV3 cells for 24 h. As shown in **Fig 15**, comparing with control cells, there was a highly significant up-regulated expression of tumor suppressor gene (*P53*) and apoptosis related genes (*Bax* and *Caspase-3*) accompanied by a significant down-regulated expression of anti-apoptosis related gene (*Bcl-2*) in the SKOV3 cells treated with FA-SnO_2_ NPs and SnO_2_ NPs, P < 0.001. Moreover, FA-SnO_2_ NPs induced higher significant alteration on apoptosis—related genes than SnO_2_ NPs in SKOV3 treated cells. For confirmation of the obtained quantitative real-time PCR results, we further investigated the levels of protein expression of these selected apoptotic genes using Immunohistochemistry and Western blotting analyses.

**Fig 16:**
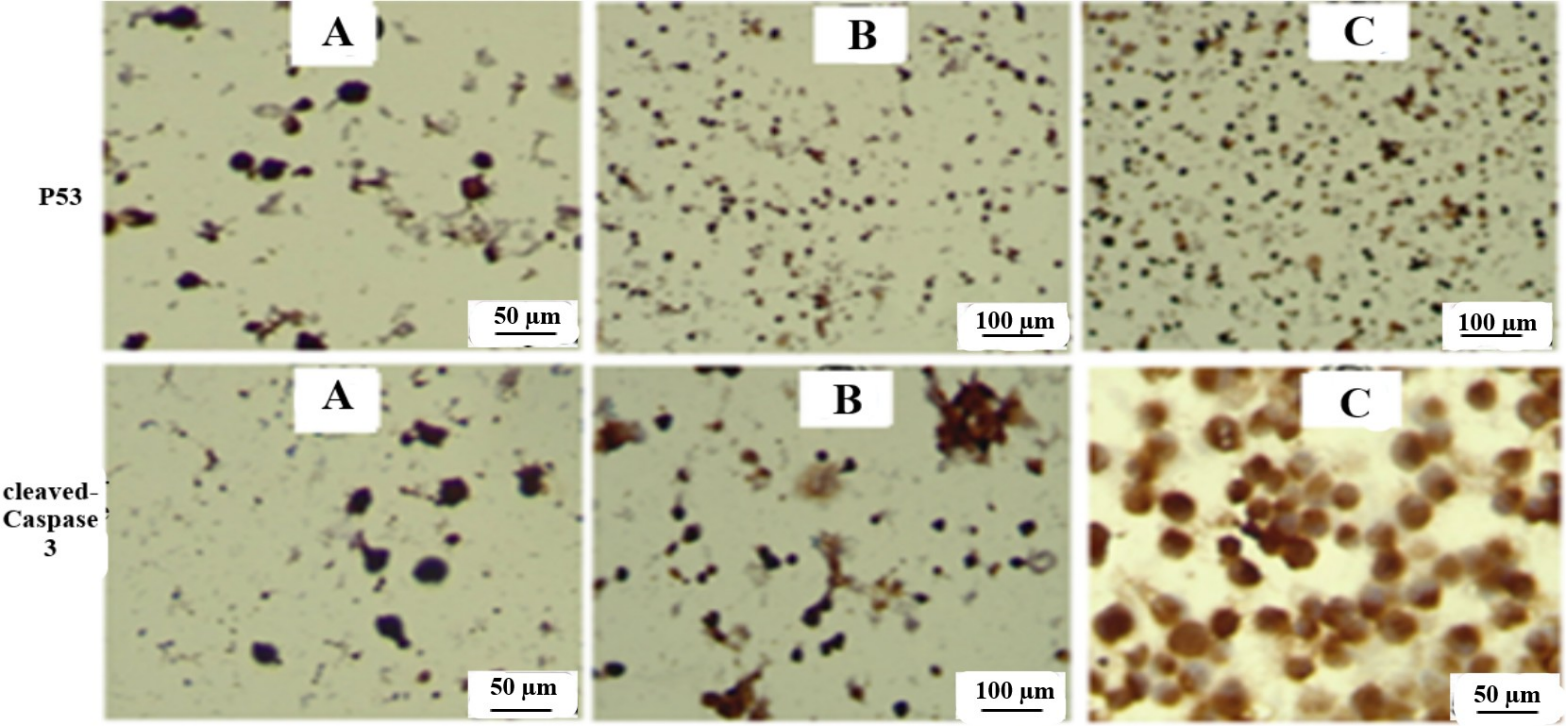
Representative photomicrographs for expression patterns of immunohistochemically markers P53 and cleaved Caspase-3. For P53: **(A)** control cells with weak staining at magnification 40×, **(B)** SnO_2_ NPs treated SKOV3 cells with moderate staining at magnification 20×, and **(C)** FA-SnO_2_ NPs treated SKOV3 cells with strong staining at magnification 20×. For cleaved Caspase-3: **(A)** control cells with weak staining at magnification 40×, **(B)** SnO_2_ NPs treated SKOV3 cells with moderate staining at magnification 20×, and **(C)** FA-SnO_2_ NPs treated SKOV3 cells with strong staining at magnification 40×.

**Fig 17:**
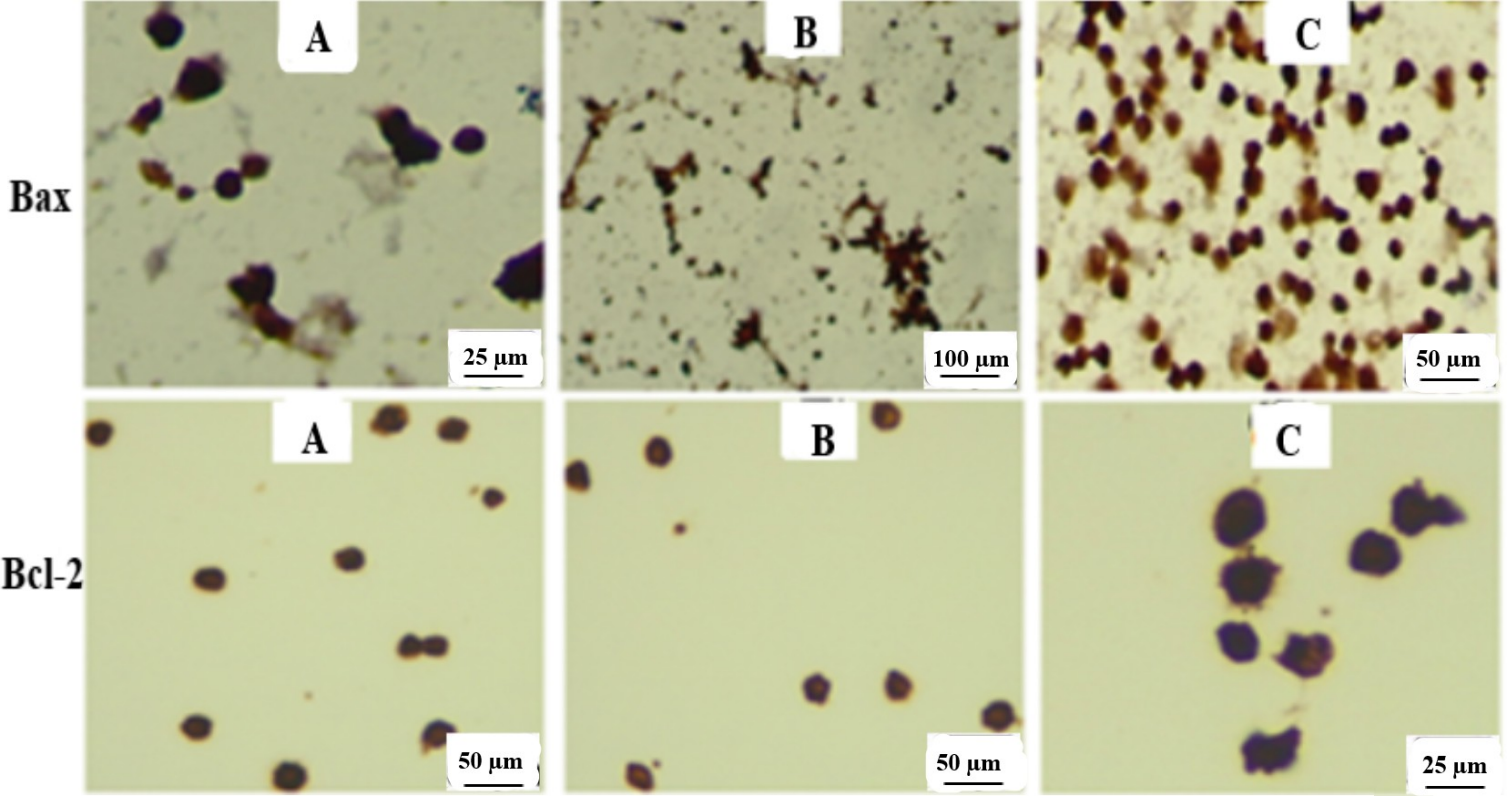
Representative photomicrographs for expression patterns of immunohistochemically markers Bax and Bcl-2. For Bax: **(A)** control cells with weak staining at magnification 60×, **(B)** SnO_2_ NPs treated SKOV3 cells with moderate staining at magnification 20×, and **(C)** FA-SnO_2_ NPs treated SKOV3 cells with strong staining at magnification 40×. For Bcl-2: **(A)** control cells with strong staining at magnification 40×, **(B)** SnO_2_ NPs treated SKOV3 cells with moderate staining at magnification 40×, and **(C)** FA-SnO_2_ NPs treated SKOV3 cells with weak staining at magnification 60×.

The effects of SnO_2_ NPs and FA-SnO_2_ NPs on the expression of P53, Bax, Bcl-2, cleaved Caspase-3 in SKOV3 treated cells via Immunohistochemistry assay are displayed in **Figs 16 and 17.** Comparing with control cells, FA-SnO_2_ NPs and SnO_2_ NPs treated SKOV3 cells showed a significant increase in the number of positive cells of P53, cleaved Caspase-3 (nuclear brown staining, **Fig 16**), and Bax **(**cytoplasmic brown staining, **Fig 17)**, accompanied with a decrease in the number of positive cells of Bcl-2 (cytoplasmic brown staining, **Fig 17)**. Furthermore, the percentages of the apoptotic index for each studied immunohistochemically marker (P53, Bax, cleaved Caspase-3, and Bcl-2) were listed in **Table 3** and the results indicated that the apoptotic index percentages of P53, Bax, and cleaved Caspase-3 in FA-SnO_2_ NPs and SnO_2_ NPs treated SKOV3 cells were significantly increased as compared to control cells, P < 0.001. These obtained data revealed FA-SnO_2_ NPs induced a higher significant alteration on apoptosis—related proteins than SnO_2_ NPs in SKOV3 treated cells, P < 0.001, that consistent with the above result of RT-PCR.

**Table 3:** Semi-quantification analysis of apoptotic cell percentages expressed as an apoptotic index (AI) by immune-staining to P53, Bax, cleaved Caspase-3, and Bcl-2 for SKOV3 treated cells with FA-SnO_2_ NPs and SnO_2_ NPs.

Immunohistochemically markers	Control cells	Apoptotic index (AI) % SnO_2_ NPs	FA-SnO_2_ NPs
**P53**	18.66 ± 0.8	46.23 ± 1.3***	82.93 ± 1.6***
**Bax**	7.23 ± 0.2	50.86 ± 0.6***	91.1 ± 0.5***
**cleaved Caspase-3**	9.3 ± 0.2	56.96 ± 1.2***	96.1 ± 1.5***
**Bcl-2**	36.23 ± 0.8	21.16 ± 3.2**	6.8 ± 0.8***

Data displayed as mean ± SD of three independent tests

***P < 0.001 and

**P < 0.01.

**Fig 18:**
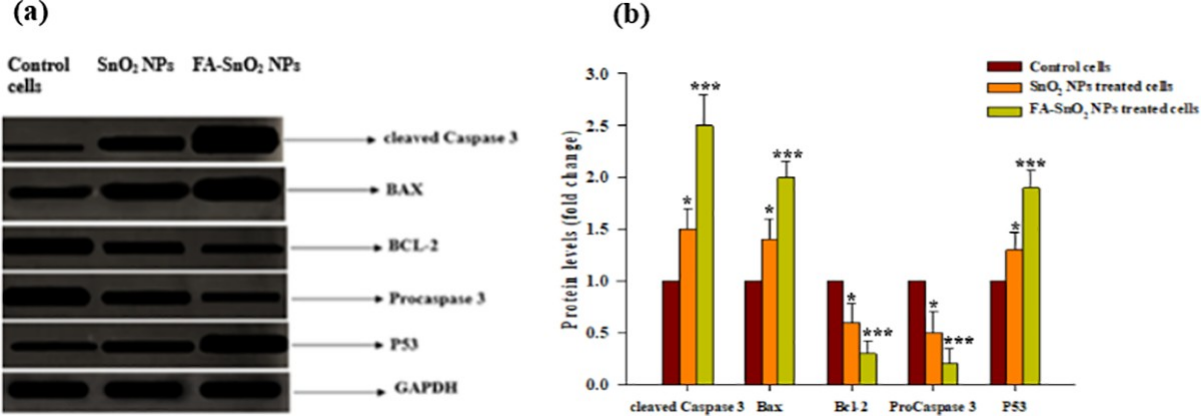
Western blotting image for protein levels of P53, proCaspase-3, Bcl-2, Bax and cleaved Caspase-3 in SnO_2_ NPs treated SKOV3 cells and FA-SnO_2_ NPs treated SKOV3 cells as compared to control cells (a), and Densitometry analysis of the fold change value from the western blot image for the studied apoptotic protein markers using AlphaEase TM FC Stand Alone V.4.0.0 software (b); *P < 0.05, **P < 0.01 and ***P < 0.001.

In line with immunohistochemically results, the obtained western blotting data for assessing the expression levels of the studied apoptotic proteins (**Fig 18A**), revealed that FA-SnO_2_ NPs had a significant stronger effect on the expression patterns of apoptotic proteins (P53, Bax, cleaved Caspase-3, proCaspase-3, and Bcl-2) than SnO_2_ NPs in SKOV3 treated cells, P < 0.001. Also, using AlphaEase TM FC StandAlone V.4.0.0 software, the estimated fold change value from the western blot image for the studied apoptotic protein markers was displayed in **Fig 18B**.

### 3.9 Toxicity effects of FA-SnO_2_ NPs to the living system using in vivo analysis

#### 3.9.1 Effect of FA- SnO_2_ NPs on hematological parameters

As can be seen in **Table 4**, there was no significant change (P > 0.05) in the measured hematological parameters (WBC, Hb, HCT, RBC, MCV, RDW and PLT) between all FA-SnO_2_ NPs treated groups comparing with the control group.

**Table 4:** Effect of different doses of FA-SnO_2_ NPs (50 mg/kg for group B, 100 mg/kg for group C and 200 mg/kg for group D) on hematological parameters of the tested male Wistar rats as compared with control group (A).

Parameters	Group (A) Control group	Group (B)	Group (C)	Group (D)	P-value
**WBC (×10** ^**3**^ **/μL)**	6.52 ± 0.81	7.84 ± 0.43	7.31 ± 0.53	7.26 ± 0.81	NS
**Hb (g/dL)**	14.3 ± 0.37	14.1 ± 0.51	13.8 ± 0.82	13.5±0.63	NS
**HCT (%)**	43 ± 0.43	42.3 ± 0.30	42.8 ± 0.61	42.5±0.38	NS
**RBC (×10** ^**6**^ **/μL)**	7.37 ± 0.24	7.04 ± 0.52	6.73 ± 0.63	6.83±0.83	NS
**MCV (fL)**	58.3 ± 0.46	59.1 ± 0.31	59.63 ± 0.85	59.84±0.95	NS
**RDW (fL)**	14.02 ± 0.48	13.37 ± 0.80	13.91 ± 0.46	13.65±0.57	NS
**PLT (×10** ^**3**^ **/μL)**	260 ± 21.03	259.81 ± 23.04	259.37 ± 24.42	259.57 ± 20.89	NS

Note: Data are expressed as mean ± SD values. Abbreviations: FA-SnO2 NPs, folic acid coated tin oxide nanoparticles; WBC, white blood cell count; Hb, hemoglobin concentration; HTC, hematocrits; RBC, red blood cell count; MCV, mean corpuscular volume; RDW, RBC distribution width; PLT, platelet count; NS, non-significant (P > 0.05).

#### 3.9.2. Effect of FA-SnO_2_ NPS on biochemical profiling

There were no significant changes (P > 0.05) in the measured biochemical markers (TLs, TP, urea, uric acid, creatinine, ALP, AST and ALT) between all FA-SnO_2_ NPs treated groups comparing with the control group (**Table 5**).

**Table 5:** Effect of different doses of FA-SnO_2_ NPs (50 mg/kg for group B, 100 mg/kg for group C and 200 mg/kg for group D) on biochemical profiling of the tested male Wistar rats as compared with control group (A).

Parameters	Group (A) Control group	Group (B)	Group (C)	Group (D)	P-value
**TLs (mg dL-1)**	470.16± 2.33	469.54± 1.78	468.87±1.55	469±1.75	NS
**TP (g dL-1)**	6.57 ± 0.05	5.81 ±1.33	5.54±0.86	5.74±0.82	NS
**Urea (mg dL-1)**	37± 0.97	38.4± 0.63	37.82±0.76	37.67±0.54	NS
**Uric acid (mg dL-1)**	2.55± 0.05	2.82± 0.67	2.94±0.35	2.77±0.93	NS
**Creatinine(mgdL-1)**	0.76± 0.30	0.78± 0.26	0.71±0.57	0. 74±0.84	NS
**ALP (IU/L)**	79.90 ± 2.33	81.32 ±1.79	80 ±1.55	80 ±2.45	NS
**AST (U L-1)**	63.4 ±1.86	62.97± 2.34	62.56±1.96	62.4±1.55	NS
**ALT (U L-1)**	27.5± 0.86	28.2± 0.67	28.4±0.83	28±0.65	NS
**CK-MB (U L-1)**	14.77± 1.33	15.85±2.31	15.24±1.67	15.55±1.49	NS

Note: Data are expressed as mean ± SD values. Abbreviations: FA-SnO_2_ NPs, folic acid-coated tin oxide nanoparticles; TLs, total lipids; TP, total proteins; ALP, alkaline phosphatase; AST, aspartate aminotransferase; ALT, alanine aminotransferase; (CK-MB), creatine kinase; NS, non-significant (P > 0.05).

#### 3.9.3 Histopathological findings for different tissues

**Fig 19:**
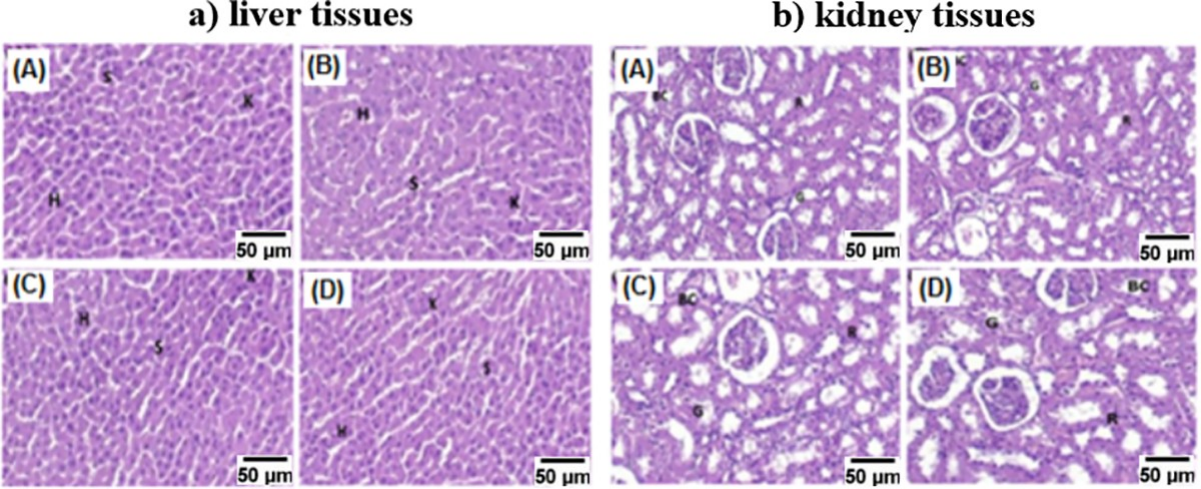
Photomicrograph of the liver (a) and kidney (b) tissues of the control group (A) and all the FA- SnO_2_ NPs treated groups (B, C, and D) of Wistar rats stained by hematoxylin and eosin dyes. **Note:** H: Hepatocytes, S: Sinusoids, K: Kupffer cells, G: Glomeruli, BC: Bowman’s capsule, and R: Renal tubule.

**Fig 20:**
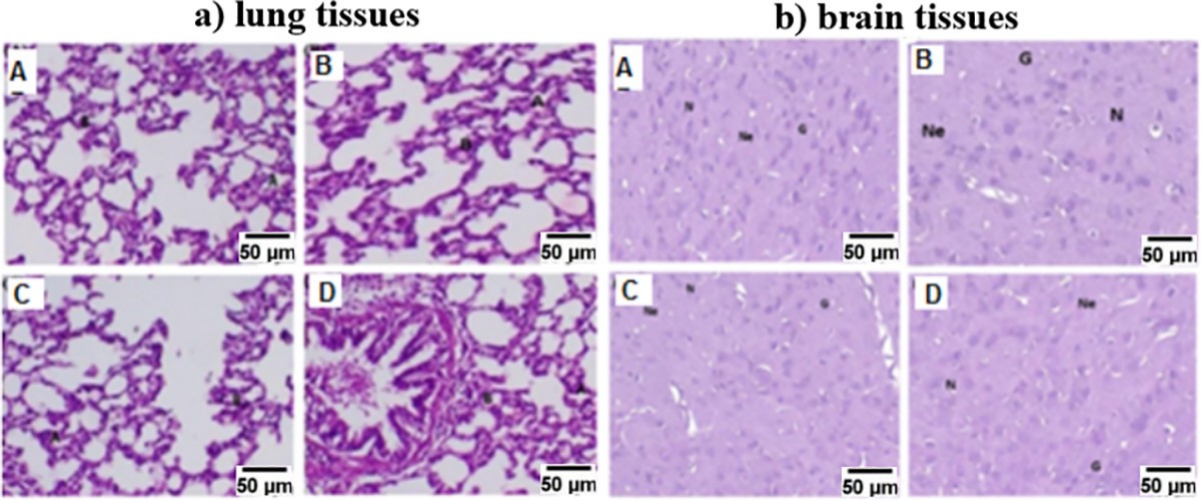
Photomicrograph of the lung (a) and brain (b) tissues of the control group (A) and all the FA-SnO_2_ NPs treated groups (B, C, and D) of Wistar rats stained by hematoxylin and eosin dyes. **Note:** B: Bronchiole, A: Alveoli, Ne: Neuropil, N: Neuronal cells and G: Glial cells.

The investigated tissues of the major vital organs for the control group (treated with saline, A) and all FA-SnO_2_ NPs treated groups (B, C, and D) using Hematoxylin and Eosin stains were displayed in **Figs 19 and 20,** showed that there wasn’t any pathological abnormalities or lesions were detected in all the FA-SnO_2_ NPs treated groups compared with the control group. The investigated tissues were as follow:

*3*.*9*.*3*.*1 Liver tissues*. The liver tissues of all tested rats groups and the control group were examined for the presence of any pathological alterations such as apoptosis, necrosis, inflammatory reactions, and fibrosis. As shown in **Fig 19A**, the investigated hepatic tissues between the control group (A) and FA- SnO_2_ NPs treated groups (B, C, and D) showed normal structures without any histological alterations between them, where the hepatocytes (H) with apparent spherical nuclei with a regular arrangement surrounded by blood sinusoids (S) with kupffer cells. So, there was no significant variation between all FA-SnO_2_ NPs treated groups comparing with the control group.

*3*.*9*.*3*.*2 Kidney tissues*. As shown in **Fig 19B**, the investigated kidney tissues of the control group (A) and FA-SnO_2_ NPs treated groups (B, C, and D) displayed normal kidney structure without any signs of segmentalization necrosis or sclerosis, the glomeruli were normocellular with exposed capillary loops and the compartments of tubular, vascular and interstitial were normal without any inflammation. Thus, there was no significant pathological change between all FA-SnO_2_ NPs treated groups in comparison with the control group.

*3*.*9*.*3*.*3 Lung tissues*. As shown in **Fig 20A**, the investigated lung tissues of the control group (A) and FA-SnO_2_ NPs treated groups (B, C, and D), displayed normal histological lung structure without any signs of lesions or inflammation, where the lung tissues have the appearance of good lace composed of thin-walled alveoli with a single layer of squamous epithelium and also, many bronchioles of various sized without any lesions or inflammation. Thus, there were no pronounced pathological differences between all FA-SnO_2_ NPs treated groups comparing with the control group.

*3*.*9*.*3*.*4 Brain tissues*. The investigated brain tissues of the control group (A) and FA-SnO_2_ NPs treated groups (B, C, and D) displayed in **Fig 20B**. The results showed a normal histological brain structure without any necrosis or inflammation, where the cerebral cortex showing a normal configuration of the cerebral capillary and normal arrangement of neuropil with intact rounded neuronal cells and glial cells (G). Thus, there was no sign of any significant pathological difference between all FA-SnO_2_ NPs treated groups comparing with the control group.

## 4. Discussion

Nanomaterials are applied in different fields of agricultural, biomedical, and environmental **[65,66]**. Recently, in biomedical field nanoparticles can be used in the active targeting of several cancer cells by its conjugation with a specific targeting therapeutic ligand for cancer surface receptors, resulting in higher accumulation of the ligand coated nanoparticles inside the cancer cells with superior toxic effects. The folate receptors are highly expressed in different cancer cells such as epithelial, ovarian, cervical, breast, and others, thus it can be used as a promising target for cancer therapy **[13].** So, our current study was designed for targeted delivery of SnO_2_ NPs to a human ovarian cancer cell line (SKOV3) using FA as a specific targeting ligand for folic acid (FA) receptors that overexpressed on ovarian cancer surface. Thus, we compared between the effects of SnO_2_ NPs and FA-SnO_2_ NPs on the cytotoxicity, oxidative stress and apoptosis induction of a human ovarian cancer cell line (SKOV3). we explored that the mechanisms of SKOV3 cell death after exposure to SnO2 NPs and FA-SnO2 NPs were likely to be mediated through ROS overproduction on time dependent manner. In addition, we explored that the mitochondria may have played a role in inducing apoptosis in SnO2 NPs and FA-SnO_2_ NPs treated cells as proved by alteration in apoptosis related proteins (upregulated expression of P53, Bax, and cleaved Caspase-3 along with downregulated of Bcl-2).

Among different chemical techniques for the preparation of SnO_2_ NPs, sol-gel method has many advantages such as well homogeneities, higher transparency, pure ashes at low temperature, and flexibility of creating nanoparticles **[16,67]**. So, in our study, we prepared SnO_2_ NPs using a sol-gel method, followed by coating with FA for preparation of FA-SnO_2_ NPs. Physicochemical characterization of nanomaterials is necessary in the research of nanotoxicity for well clarification of results **[68].** Characterization of SnO_2_ NPs and FA-SnO_2_ NPs were done by different techniques. Firstly, the obtained results using the DLS technique showed that the average particle size of the uncoated SnO_2_ NPs was about 151 nm and increased to 157 nm after coating with FA (FA-SnO_2_ NPs). The increment of the SnO_2_ NPs size after FA coating can be attributed to an increase in the shell volume caused by FA conjugation onto the SnO_2_ NPs surface. The Zeta potential of the SnO_2_ NPs was found to be +15.8 mV and changed to a negative value after coating the SnO_2_ NPs with FA (-24 mV). This may be due to the presence of residual free carboxylic acid groups (−COO^–^) of FA. Similar findings were previously mentioned for shifting the negative values of the zeta potential toward the positive one for different nanoparticles after FA conjugation **[15,69–71]**.

Secondly, the conjugation of SnO_2_ NPs with FA was also confirmed by FT-IR, UV-visible, and XRD analyses. The FA displayed a characteristic absorption band around 289 nm corresponded to C–C bond transition (л-л*) and another band around 360.5 nm assigned to the C–O bond transition (n-л*) **[72].** Comparison of FT-IR spectra of uncoated and coated SnO2 NPs showed some modification of the characteristic peaks of SnO_2_ NPs alone (at 1249 cm−1, 594 cm−1, 501 cm−1, and 439 cm−1) and the characteristic peak due to carboxylic group of FA, suggesting the modification of SnO2 NPs surface by combination of FA. These findings were consistent with several previous reports **[22,73]**. UV-visible spectrum of SnO_2_ NPs coated with FA, however, exhibited a characteristic peak of the SnO_2_ NPs at 219 nm, which overlapped with the two characteristic adsorption peaks for FA at 284 nm and 362 nm, suggesting successfully coated FA ligand onto SnO_2_ NPs surface.

Previous studies reported that the metal oxide nanoparticles showed sharp diffraction peaks with good crystalline character, and decreasing their size resulted in peak broadening after coating **[74].** Also, the reduction in crystallinity resulted in a decrease the thermodynamic stability along with increasing dissolution rate and bioavailability of well-dispersed nanoparticles **[75]**. Similar results were observed from our XRD measurement. It can be estimated that the average size of SnO_2_–NPs lie nearly around 110 nm for SnO_2_–NPs nm and 53 nm for coated SnO_2_–NPs one. However, the size estimate of nanoparticles from Scherrer method lower than DLS, but it may be a rough estimate from the D values in the Scherrer’s formula.

Finally, the morphological analysis of the prepared SnO_2_ NPs and FA-SnO_2_ NPs surfaces, as examined with SEM and TEM, revealed that the presence of FA decreased the average of SO_2_ NPs from 74 to 49, irrespective of shape. The higher size of our prepared SnO_2_ NPs and FA-SnO_2_ NPs nanoparticles suspension, determined from LDS technique in aqueous, comparing to the size obtained from XRD and TEM measurements might be as a result of affinity of these particles for agglomeration in aqueous state **[76]**. Thus, from the obtained data, it can be concluded that the SnO_2_ NPs are successfully coated with FA.

Generally, it was reported that the size, charge, shape, and surface coating of NPS, may cause changes in its therapeutic efficacy, where the average particle size and distribution of nanoparticles mostly depend on the relative degrees of growth, nucleation processes, and the level of agglomeration, so the size of the fine particle of nanoparticles with the large surface area may increase its bioavailability and therapeutic efficiency in cancer treatment **[3]**. Therefore, one of the main goals of the present current study was to study the cytotoxic effect of SnO_2_ NPs and FA-SnO_2_ NPs against the SKOV3 ovarian cancer cells.

The proliferation of SKOV3 cancer cells using MTT assay has shown a characteristic concentration-dependent cytotoxic effect of SnO_2_ NPs and FA-SnO_2_ NPs in SKOV3 cancer cells. As the concentrations of SnO_2_ NPs, as well as SnO_2_ NPs, increased, the viability rate of SKOV3 cells was significantly decreased along 24 h. Besides, FA-SnO_2_ NPs displayed a higher anti-proliferative effect with more effective IC50 concentration against SKOV3 cells than SnO_2_ NPs. This finding may be due to specific interaction between FA and the folate receptors that are overexpressed on the cancer cells and less or no expression was found on healthy cells **[15]**, where the FA-SnO_2_ NPs will interact with SKOV3 cancer cells and then internalized by the mechanism of receptor-mediated endocytosis, resulting in entering higher concentration of FA-SnO_2_ NPs inside the cancer cells and potent toxic effect against SKOV3 cells with less toxicity on healthy cells. Moreover, using microscopic examination, FA-SnO_2_ NPs showed severe morphologic alterations of SKOV3 cells damage than SnO_2_ NPs comparing with control cells. This observation was in agreement with the obtained cytotoxicity results that revealed higher intracellular uptake of FA-SnO_2_ NPs.

Also, measurement of LDH activity is considered to be an additional indicator for cell viability via assessment of the permeability of cell membrane. Consequently, the obtained data for FA-SnO_2_ NPs was showed higher percentage of LDH leakage than SnO_2_ NPs in treated SKOV3 cells. Previous studies suggested there was a relationship between the direct association of LDH enzyme upregulation and successive apoptosis induction **[3]**. Thus, the prepared FA-SnO_2_ NPs is considered to be an effective treatment for ovarian cancer. Similar observations of the anticancer activities of the SnO_2_ NPs against different human cancer cell lines other than ovarian cancer were previously reported **[23–26]**. Also, previous studies were reported the cytotoxic effects of metal-metal oxide nanoparticles against different cancer cells other than SnO_2_ NPs such as selenium nanoparticles **[77],** cerium oxide nanoparticles **[78]**, and magnesium oxide nanoparticles **[79].**

The differentiation between cell death mechanisms is essential for the detection of the type of cell cytotoxic pathway (apoptosis or necrosis). Apoptosis pathway is a form of programmed cell death that is genetically controlled for regulatory tissue growth via an organized physiological means for removing physical damage and abnormal cells **[80].** So, in our study, different assays for detection and progression of apoptosis induction were evaluated such as Annexin V-FITC/PI, AO/EB staining, and Comet assays. Consequently, the obtained cytometry results using the Annexin V-FITC/PI technique confirmed the apoptosis induction in SKOV3 cells under the effect of the SnO_2_ NPs and FA-SnO_2_ NPs. Consistent with MTT assay, FA-SnO_2_ NPs exposed a significant effect on the SKOV3 apoptosis (early and late) than SnO_2_ NPs. The high effect of the apoptotic cells in the SKOV3 treated with the FA-SnO_2_ NPs may be due to the increase of the intracellular uptake of FA-SnO_2_ NPs resulted in a superior therapeutic effect. This finding was supported by a previous study for FA-titanium NPs **[15]**.

Furthermore, apoptosis progression in SKOV3 cells is also confirmed using AO/EB assay. Detection of the nuclear morphological alterations of cell membranes throughout apoptosis along with differentiation among altered cell types (viable cells, necrotic cells, and early and late apoptotic cells), were investigated using AO/EB staining under the fluorescence microscope. The obtained results for the effect of the SnO_2_ NPs and FA-SnO_2_ NPs on nuclei of SKOV3 cells indicated that a higher significant effect of the early and late apoptotic cells in the SKOV3 treated with FA-SnO_2_ NPs compared with SnO_2_ NPs, which was in covenant with the other obtained Annexin V-FITC/PI staining results.

The cleaved Caspases 3 (activated Caspase3) has an important role in cell death mediated apoptosis causing nuclear condensation and DNA damage **[81]**. So, the DNA damage in SKOV3 cells treated with SnO_2_ NPs and FA-SnO_2_ NPs owing to apoptosis induction was examined using comet assay. The obtained results using OTM parameter (the finest parameter of comet assay that is used for calculation of DNA damage in apoptotic cells), showed a significantly higher level of DNA damage in SKOV3 cells treated with FA-SnO_2_ NPs than SnO_2_ NPs. Moreover, FA-SnO_2_ NPs treated cells showed a higher intensity of comet tails than SnO_2_ NPs treated one. This finding again confirmed the potent synergistic influence of FA-SnO_2_ NPs towards SKOV3 cancer cells.

For further detection of the mechanisms that related to the cytotoxic effects of FA-SnO_2_ NPs and SnO_2_ NPs on SKOV3, we measured the percentages of SKOV3 cells existing in each cell cycle phase using flow cytometry. The cells that have damaged DNA due to apoptosis induction will accumulate resulting in arresting the cell cycle in G1 (gap1), S (DNA synthesis), or in G2/M (gap2/mitosis) phase **[82]**. Our results showed highly significant increment of apoptotic cells percentage in the G2/M and sub G1 phases of FA-SnO_2_ NPs treated cells than SnO_2_ NPs treated cells. This may be attributed to the high accumulation of FA-SnO_2_ NPs inside SKOV3 cells via reorganization of FA by a folic acid receptor that overexpressed on SKOV3 surface. This finding was consistent with results reported previously of TiNPs coated with FA that were recognized by osteosarcoma cells **[15]**. Also, it has been suggested that ROS and P53 were mediated in induction of cell cycle arrest and apoptosis **[83].** So the obtained results of arresting SKOV3 cells in G2/M phase after treatment with FA-SnO_2_ NPs and SnO_2_ NPs maybe related to activation of P53 protein that mediated of apoptosis induction after ROS overproduction and DNA damage. Previous results demonstrated that SnO_2_ NPs induced cell cycle arrest in human MCF-7 cells due to apoptosis induction **[25].**

Oxidative stress is considered to be one of the most significant toxicity mechanisms of nanoparticles. The excessive generation of ROS in NPs treated cancer cells is due to the small size and large surface area of NPs, resulting in several cellular events including inflammation, DNA damage and finally cell apoptosis **[84]**. In our current study, we found that FA-SnO_2_ NPs were more efficient in the production of ROS as compared to SnO_2_ NPs in SKOV3 treated cells on a time-dependent mode. This suggested that oxidative stress is an essential mediator of cytotoxicity induced by SnO_2_ NPs and FA-SnO_2_ in SKOV3 cells. Similar observation of the overproduction of ROS on time dependent manner in oral cancer cells after treatment with SnO_2_ NPs was previously reported **[23]**. However, other previous study was reported that the effect of SnO_2_ NPs against human breast cancer cells was through intracellular ROS increment in dose dependent manner **[25]**.

It has been reported that Bcl-2 family have regulators proteins for apoptosis, where their functions are either pro-apoptotic (Bax, Bak and Bad) or anti-apoptotic (Bcl-2 and Bcl-XL) regulators **[29]**. So, in the current study, we evaluated the effects of SnO_2_ NPs and FA-SnO_2_ NPs on the expression levels of mRNA of some apoptotic genes coding for apoptosis related proteins (*P53*, *Bax*, *Caspase-3*, and *Bcl-2*) using quantitative RT-PCR technique and confirmed the results with investigation the levels of protein expression of these selected apoptosis related genes in SKOV3 cells using Immunohistochemistry and Western blotting analyses.

Oxidative stress and DNA damage can stimulate activation of tumor suppressor protein (P53), resulting in apoptosis induction mediated by the activated p53 protein through transcription-dependent mechanism, where P53 has an important role in upregulating Bax expression; that is the key intrinsic way of apoptosis **[85]**. In the present study, we found that the expressions of both mRNA and protein levels of P53 and Bax were up-regulated whereas the expression of Bcl-2 was downregulated in SKOV3 cells treated with SnO_2_ NPs and FA-SnO_2_ NPs. This finding suggests that Bax is up-regulated by P53, resulting in P53-mediated apoptosis in SKOV3 cells. Moreover, upregulation of Bax induces the mitochondrial permeabilization, resulted in releasing higher levels of cytochrome c into the cytosol, which stimulate Caspase-3 activation **[81]**. Consequently, the activated Caspase-3 leads to DNA damage and execution of apoptosis along with inhibition of the expression levels of BCL-2 gene **[86].** This finding was in line with our obtained data that showed higher expression level of activated Caspase 3 protein in SKOV3 cells treated with SnO_2_ NPs and FA-SnO_2_ NPs, suggesting that Caspase 3-mediated apoptosis in SKOV3 cells. This finding was confirmed by our above obtained data for DNA damage that was observed in SKOV3 cells treated with SnO_2_ NPs and FA-SnO_2_ NPs using comet assay.

From all above obtained results, we conclude that the FA-SnO_2_ NPs had a significant induced cytotoxicity in SKOV3 cancer cells in dose-dependent mode through generation of higher levels of ROS. The ROS-mediated cell apoptosis through intrinsic mitochondrial pathway resulted in P53-mediated apoptosis and Caspase 3-dependent cell death.

Examination of the effects of tagged nanomaterials on normal cellular function is essential for confirmation the safety of using these tagged nanomaterials in therapeutic applications **[87]**. So, we have investigated the effects of our prepared FA-SnO_2_ NPs against living system using in vivo studies for confirmation of the safety of its utilization, where FA-SnO_2_ NPs were intraperitoneally administered to the tested Wistar rat and then some toxicological parameters such as hematological, biochemical and histopathological were evaluated. The obtained results revealed that FA-SnO_2_ NPs have not triggered any toxicity to the main organs for the tested tissues. Therefore, from the obtained data, it can be concluded that the FA-SnO_2_ NPs can be used as a safe therapeutic agent for treatment human ovarian cancer.

## 5. Conclusions

Our current study suggested a beneficial insight into the cellular and molecular events involved in the ovarian cancer cell apoptosis caused by FA-SnO_2_ NPs at in vitro level. Here, the obtained data showed that coating of SnO_2_ NPs surface with folic acid, which acts as a specific targeting ligand for SKOV3 cancer cells resulted in a highly significant cytotoxic effects than uncoated SnO_2_ NPs. Moreover, all the investigation mechanisms for the apoptosis induction in the treated SKOV3 cells showed the superior apoptosis levels of SKOV3 cells after treatment with FA-SnO_2_ NPs, that were likely to be mediated through ROS overproduction on time dependent manner. In addition, FA-SnO_2_ NPs induced a higher significant alteration on apoptosis- related proteins (upregulation expression P53, Bax, and cleaved Caspase-3 along with decreasing of Bcl-2 levels), which suggest that apoptosis induction may have occurred through mitochondrial pathway within SKOV3 cells. Ultimately, the P53 and the activated Caspases-3 are responsible for SKOV3 cell death mediated apoptosis. Over all, the superior anticancer effect of our prepared FA-SnO_2_ NPs along with the safety utilization in the biomedical applications which was confirmed by in vivo studies could potentially be used as a potent chemotherapeutic agent for ovarian cancer treatment. However, the potent cytotoxic effect of FA-SnO_2_ NPs within SKOV3 cell was through short-term exposure, so additional long-term exposure, extensive animal experiments, and clinical trials are needed.

## Abbreviations

FTIR: Fourier-transform infrared spectroscopy
UV-vis spectroscopy: Ultraviolet-visible spectroscopy
XRD: X-Ray Diffraction
SEM: Scanning electron microscope
TEM: Transmission electron microscope
MTT: 3-(4,5-Dimethylthiazol-2-yl)-2,5-diphenyltetrazolium bromide
Annexin V/PI: Annexin V-propidium iodide PI
AO/EB: Acridine orange- ethidium bromide
FA: Folic acid
qRT-PCR: Quantitative real time- polymerase chain reaction
